# Chemical strategies to modify amyloidogenic peptides using iridium(iii) complexes: coordination and photo-induced oxidation†
†Electronic supplementary information (ESI) available: Experimental section and Fig. S1–S19. See DOI: 10.1039/c9sc00931k


**DOI:** 10.1039/c9sc00931k

**Published:** 2019-06-05

**Authors:** Juhye Kang, Jung Seung Nam, Hyuck Jin Lee, Geewoo Nam, Hyun-Woo Rhee, Tae-Hyuk Kwon, Mi Hee Lim

**Affiliations:** a Department of Chemistry , Korea Advanced Institute of Science and Technology (KAIST) , Daejeon 34141 , Republic of Korea . Email: miheelim@kaist.ac.kr; b Department of Chemistry , Ulsan National Institute of Science and Technology (UNIST) , Ulsan 44919 , Republic of Korea . Email: kwon90@unist.ac.kr; c Department of Chemistry Education , Kongju National University , Gongju 32588 , Republic of Korea; d Department of Chemistry , Seoul National University , Seoul 08826 , Republic of Korea

## Abstract

Amyloidogenic peptides are considered central pathological contributors towards neurodegeneration as observed in neurodegenerative disorders [*e.g.*, amyloid-β (Aβ) peptides in Alzheimer's disease (AD)]; however, their roles in the pathologies of such diseases have not been fully elucidated since they are challenging targets to be studied due to their heterogeneous nature and intrinsically disordered structure. Chemical approaches to modify amyloidogenic peptides would be valuable in advancing our molecular-level understanding of their involvement in neurodegeneration. Herein, we report effective chemical strategies for modification of Aβ peptides (*i.e.*, coordination and coordination-/photo-mediated oxidation) implemented by a single Ir(iii) complex in a photo-dependent manner. Such peptide variations can be achieved by our rationally designed Ir(iii) complexes (**Ir-Me**, **Ir-H**, **Ir-F**, and **Ir-F2**) leading to significantly modulating the aggregation pathways of two main Aβ isoforms, Aβ_40_ and Aβ_42_, as well as the production of toxic Aβ species. Overall, we demonstrate chemical tactics for modification of amyloidogenic peptides in an effective and manageable manner utilizing the coordination capacities and photophysical properties of transition metal complexes.

## Abstract 2

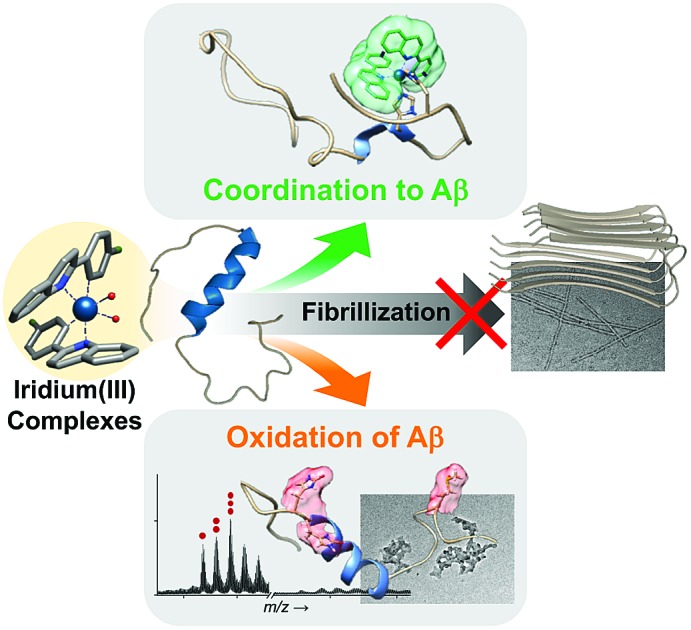
Effective chemical strategies, *i.e.*, coordination and coordination-/photo-mediated oxidation, are rationally developed towards modification of amyloidogenic peptides and subsequent control of their aggregation and toxicity.

## Introduction

A substantial amount of research effort has been dedicated towards identifying the association of amyloidogenic peptides with the pathologies of neurodegenerative diseases. Among these amyloidogenic peptides, amyloid-β (Aβ), a proteolytic product of the amyloid precursor protein found in the AD-affected brain with a self-aggregation propensity, has been implicated as a pathological factor in Alzheimer's disease (AD).^1^–^4^ As the main component of senile plaques, Aβ accumulation is a major pathological feature of AD.^1^–^3^,^5^ Recent developments in Aβ research (*e.g.*, clinical failures of Aβ-directed therapeutics) have led to the re-evaluation of the amyloid cascade hypothesis.^6^ Aβ pathology, however, remains a pertinent facet of the disease with indications of Aβ oligomers as toxic species responsible for disrupting neuronal homeostasis.^1^–^3^,^7^ Furthering our elucidation of Aβ pathology presents an investigative challenge arising from its heterogeneous nature and intrinsically disordered structure.^1^,^2^ To overcome this obstacle and advance our understanding of the Aβ-related contribution towards AD, in this study, we illustrate chemical approaches to modify Aβ peptides at the molecular level using transition metal complexes.

**Fig. 1:**
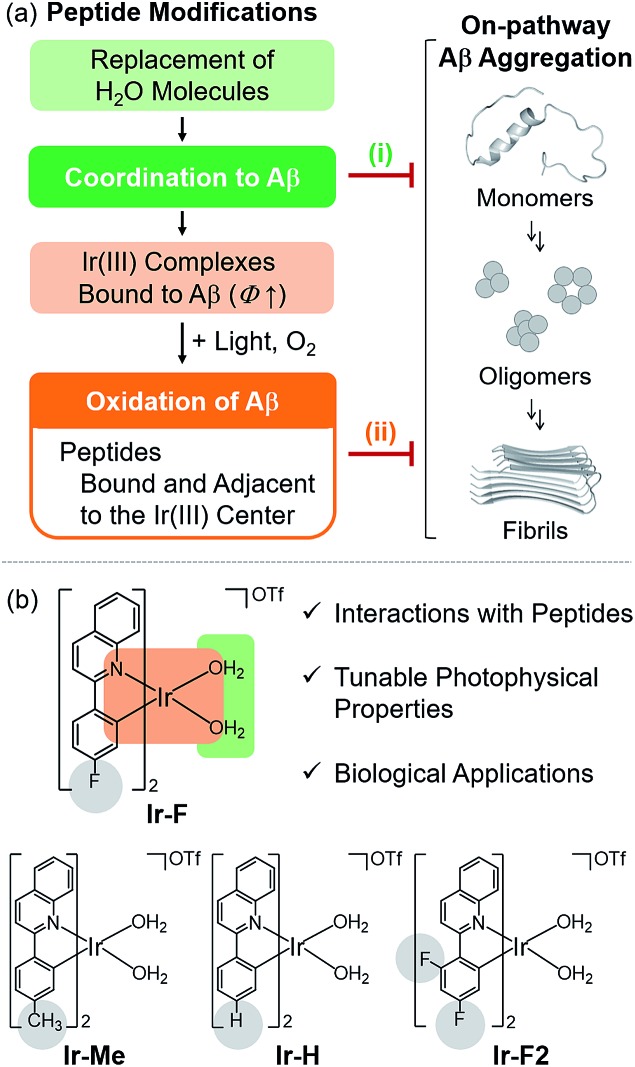
Chemical approaches to modifying Aβ peptides using rationally designed Ir(iii) complexes. (a) Two events of modifying Aβ peptides using Ir(iii) complexes for controlling of Aβ aggregation: (i) coordination to Aβ peptides and (ii) oxidation of Aβ peptides mediated by coordination and photoactivation (*Φ* = emission quantum yield). (b) Design criteria and chemical structures of **Ir-F**, **Ir-Me**, **Ir-H**, and **Ir-F2**. Substituents are highlighted in gray.

Transition metal complexes have been reported to harness their ability to induce peptide modifications (*e.g.*, hydrolytic cleavage and oxidation), inhibit the activities of enzymes, and image cellular components.^8^–^43^ In particular, the ability of transition metal complexes to alter peptides stems from their properties, such as the capacity for peptide coordination.^17^–^29^,^36^,^37^ Herein, we report effective chemical strategies for modification of Aβ peptides using a single Ir(iii) complex in a photo-dependent manner (Fig. 1). Aβ modifications, achieved by our rationally engineered Ir(iii) complexes, include two events: (i) complexation with Aβ in the absence of light; (ii) Aβ oxidation upon coordination and photoactivation, which can significantly regulate their aggregation and toxicity. Through our multidisciplinary studies, presented in this work, we demonstrate the development of new chemical tactics for modification of amyloidogenic peptides using transition metal complexes, useful for identifying their properties, such as aggregation, at the molecular level.

## Results and discussion

### Rational strategies for peptide modification using Ir(iii) complexes

**Scheme 1:**
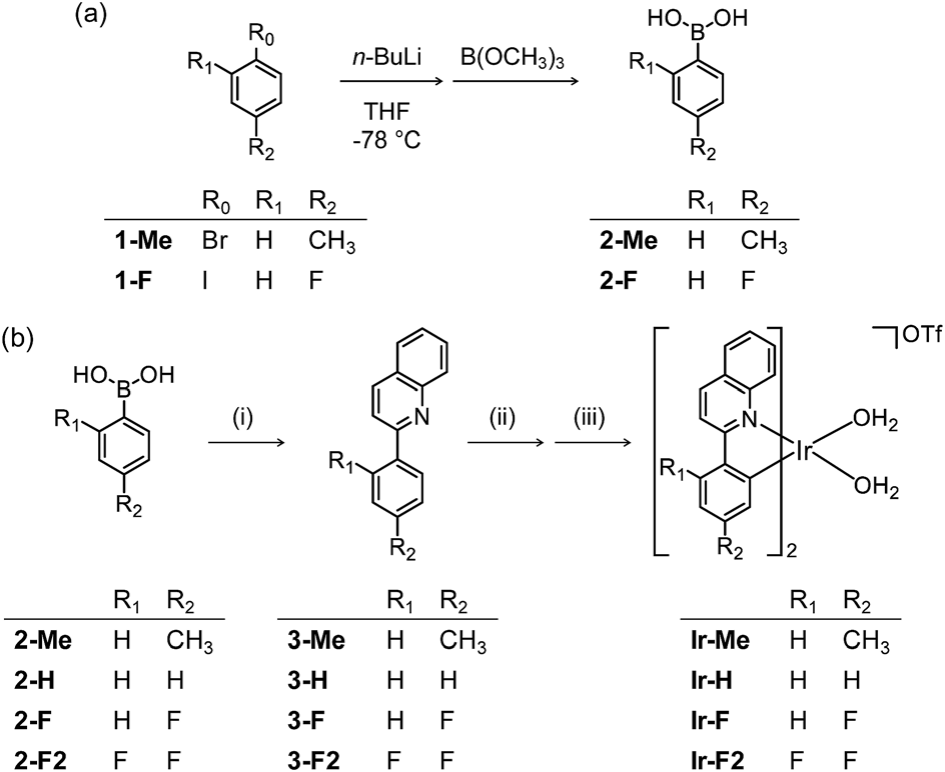
Synthetic routes to Ir(iii) complexes. Reagents and conditions: (i) 2-chloroquinoline, Pd(PPh_3_)_4_, THF/K_2_CO_3_ (aq), Δ; (ii) IrCl_3_·*n*H_2_O, 2-methoxyethanol/H_2_O, Δ; (iii) AgOTf, CH_3_OH/CH_2_Cl_2_.

To chemically modify Aβ peptides in a photoirradiation-dependent manner (Fig. 1a), four Ir(iii) complexes (**Ir-Me**, **Ir-H**, **Ir-F**, and **Ir-F2**; Fig. 1b) were rationally designed and prepared. Iridium is a third row transition metal exhibiting strong spin–orbit coupling at the center of Ir(iii) complexes with facile electronic transitions.^44^,^45^ This spin–orbit coupling can be further strengthened by fine-tuning the ancillary ligands of Ir(iii) complexes. As a result, Ir(iii) complexes confer notable photophysical properties upon excitation by relatively low energy irradiation in the visible range, including their ability to generate reactive oxygen species [ROS; *e.g.*, singlet oxygen (^1^O_2_) and the superoxide anion radical (O_2_˙^–^)] *via* electron or energy transfer.^46^–^48^ In addition, Ir(iii) complexes with octahedral geometry are relatively stable upon light activation.^48^ Incorporation of 2-phenylquinoline derivatives as ligands yielded high emission quantum yield (*Φ*) and robust ROS generation.^46^ Therefore, ancillary ligands of four complexes were constructed based on the 2-phenylquinoline backbone by applying simple structural variations to provide appropriate structural and electronic environments to promote the photochemical activity of the corresponding Ir(iii) complexes.^46^ Moreover, fluorine atoms were introduced into the ancillary ligand framework affording **Ir-F** and **Ir-F2** to chemically impart the ability to interact with Aβ through hydrogen bonding, alter photophysical properties of the complexes, and enhance the molecules' biocompatibility.^49^–^51^ Two water (H_2_O) molecules were incorporated as ligands to enable covalent coordination to Aβ *via* replacement with amino acid residues of the peptide, *e.g.*, histidine (His).^20^,^52^,^53^ The four Ir(iii) complexes were synthesized following previously reported procedures with modifications (Scheme 1 and Fig. S1–S3†).^20^,^54^–^56^ As depicted in Fig. S4 and S5,† these Ir(iii) complexes were confirmed to coordinate to His or Aβ in both H_2_O and an organic solvent [*i.e.*, dimethyl sulfoxide (DMSO)] under our experimental conditions.

### Coordination-dependent photophysical properties and ROS production of Ir(iii) complexes

Photophysical properties of the prepared Ir(iii) complexes were investigated by UV-vis and fluorescence spectroscopy. As shown in Table 1 and Fig. S6,† in the absence of His or Aβ, low *Φ* values of the four Ir(iii) complexes were observed, along with relatively poor ^1^O_2_ generation with photoactivation. Note that a solar simulator (Newport IQE-200) was used to irradiate the samples at a constant intensity (1 sun light; 100 mW cm^–2^). Upon addition of His, the *Φ* values of the four Ir(iii) complexes drastically increased (*e.g.*, *Φ***_Ir-F_** = 0.0071 *versus Φ***_Ir-F_**_+His_ = 0.26; Table 1), indicating His coordination of the complexes, which was further confirmed by electrospray ionization-mass spectrometry (ESI-MS) (Fig. S5a†). The *Φ* values and ^1^O_2_ formation of the four Ir(iii) complexes with His binding exhibited trends similar to their binding affinity with His (**Ir-F** > **Ir-H** > **Ir-Me** > **Ir-F2**). **Ir-F**, indicating the strongest binding affinity with His (Fig. S5b†), among the four Ir(iii) complexes, showed notable binding affinities towards different Aβ species (for monomers, *K*_d_ = 1.6 × 10^–4^ M; for oligomers, *K*_d_ = 2.6 × 10^–4^ M; for fibrils, *K*_d_ = 7.1 × 10^–4^ M; Fig. S7†). **Ir-F**, exhibiting a relatively high value of *Φ* upon His binding, also produced significant amounts of ^1^O_2_ and O_2_˙^–^ in the presence of His with photoactivation (Fig. S6 and S8†). Based on these properties, we selected **Ir-F** as a representative candidate of our Ir(iii) complexes and illustrated its ability to modify Aβ peptides in detail (*vide infra*).

**Table 1:** Photophysical properties of Ir(iii) complexes

	**Ir-Me**		**Ir-H**		**Ir-F**		**Ir-F2**	
	–His	+His	–His	+His	–His	+His	–His	+His
*λ*_ex,max_ (nm), (*ε*, ×10^3^ M^–1^ cm^–1^)	280 (±27), 336 (±14), 449 (±3)	274 (±29), 343 (±16), 449 (±4)	275 (±29), 339 (±15), 452 (±3)	268 (±32), 338 (±15), 445 (±3)	274 (±34), 336 (±17), 446 (±4)	269 (±33), 335 (±16), 433 (±4)	277 (±36), 350 (±21), 441 (±7)	272 (±37), 348 (±19), 432 (±6)

*λ*_em,max_ (nm)	587	592	587	593	589	573	573	578

*Φ*	0.0038 (±0.0007)	0.19 (±0.01)	0.0037 (±0.0006)	0.31 (±0.03)	0.0071 (±0.001)	0.26 (±0.03)	0.0027 (±0.0002)	0.081 (±0.007)

*τ* (ns)	5.8 (±1.8)	601 (±20)	11 (±1)	619 (±61)	4.8 (±2.0)	810 (±23)	4.4 (±0.4)	484 (±41)

*k*_r_ (×10^5^ s^–1^)	6.5	3.2	3.3	5.0	15	3.3	6.1	1.7

*k*_nr_ (×10^5^ s^–1^)	1.7 × 10^3^	13	0.88 × 10^3^	11	2.1 × 10^3^	9.1	2.2 × 10^3^	19

### Photoirradiation-dependent peptide modification using Ir(iii) complexes

**Fig. 2:**
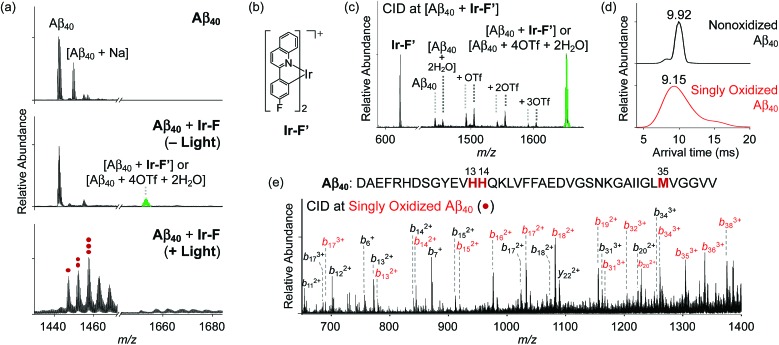
Analysis of Aβ_40_ species generated upon treatment with **Ir-F**. (a) ESI-MS spectra of **Ir-F**-incubated +3-charged Aβ_40_ with and without light. The peak indicated in green corresponds to a complex of Aβ_40_ and **Ir-F′** [structure shown in (b)]. The peaks corresponding to oxidized Aβ_40_ species are indicated with red dots. The number of red dots represents the number of oxygen atoms incorporated into Aβ_40_. (c) Collision-induced dissociation (CID) spectrum at 1653 *m*/*z* [green peak from (a)]. (d) Arrival time distributions (ATDs) between nonoxidized and singly oxidized Aβ_40_ monomers. (e) Sequence of Aβ_40_ and CID spectrum of the singly oxidized Aβ_40_ found in (a). Monooxidized *b* fragments are denoted in red. Charges are omitted in the MS spectra. Conditions: [Aβ_40_] = 100 μM; [**Ir-F**] = 500 μM; 37 °C; 1 h; no agitation; 1 sun light for 10 min (for the samples treated with light); aerobic conditions.

Modification of Aβ peptides upon treatment with **Ir-F** was monitored *via* mass spectrometric techniques [*i.e.*, ESI-MS, ESI-MS^2^, and ion mobility-mass spectrometry (IM-MS)]. The ESI-MS analysis of **Ir-F**-treated Aβ samples revealed the complex formation between Aβ and **Ir-F′** [the **Ir-F** form that does not have two H_2_O molecules bound to the Ir(iii) center; Fig. 2b] in the absence of light as an indication at 1653 *m*/*z* (Fig. 2a, middle; green). To identify the molecular species corresponding to 1653 *m*/*z*, the peak was further analyzed *via* ESI-MS^2^ in conjunction with collision-induced dissociation (CID; Fig. 2c). The detected ion fragments exhibited *m*/*z* values attributed to Aβ_40_ and **Ir-F′**. Therefore, our MS results demonstrate the complexation between Aβ and **Ir-F** with the loss of two H_2_O molecules from the Ir(iii) center (**Ir-F′**; Fig. 2b). Note that the *m*/*z* value of the **Ir-F′**–Aβ_40_ complex is equal to that of [Aβ_40_ + 4OTf + 2H_2_O]; thus, we cannot rule out the co-existence of the complex and an OTf adduct.

Upon photoirradiation, the ESI-MS analysis of **Ir-F**-treated Aβ_40_ samples led to the detection of oxidized Aβ_40_ (Fig. 2a, bottom). Aβ_40_ oxidation manifested a conformational change as probed by IM-MS (Fig. 2d). The most dominant arrival time indicated a peak at 9.92 ms. These results suggest that Aβ_40_ oxidation induced by **Ir-F** can alter the structural distribution of Aβ_40_. Similar observations were observed with **Ir-Me**, **Ir-H**, and **Ir-F2**, where the complexes were able to oxidize Aβ_40_ and consequently vary its structural distribution (Fig. S9 and S10†). In order to determine the location of peptide oxidation, the Aβ fragment ions, generated by selectively applying collisional energy to singly oxidized Aβ, were analyzed by ESI-MS^2^ (Fig. 2e). All *b* fragments smaller than *b*_13_ were detected in their nonoxidized forms, while those larger than *b*_34_ were only monitored in their oxidized forms. The *b* fragments between *b*_13_ and *b*_34_ were indicated in both their oxidized and nonoxidized forms. Such observations, along with previous reports regarding Aβ oxidation,^19^,^57^ suggest His13, His14, and Met35 of Aβ as plausible oxidation sites. Collectively, our studies demonstrate that Aβ peptides can be modified upon treatment with **Ir-F** [(i) coordination to Aβ by replacing two H_2_O molecules with the peptide in the absence of light; (ii) coordination-mediated oxidation of Aβ at three possible amino acid residues (*e.g.*, His13, His14, and Met35) upon photoactivation (Fig. 1a)]. Note that the Aβ samples produced by treatment of photoactivated **Ir-F** showed high fluorescence intensity and were relatively stable in both H_2_O and cell growth media (Fig. S11†).

### Effects of peptide modifications triggered by Ir(iii) complexes on Aβ aggregation

**Fig. 3:**
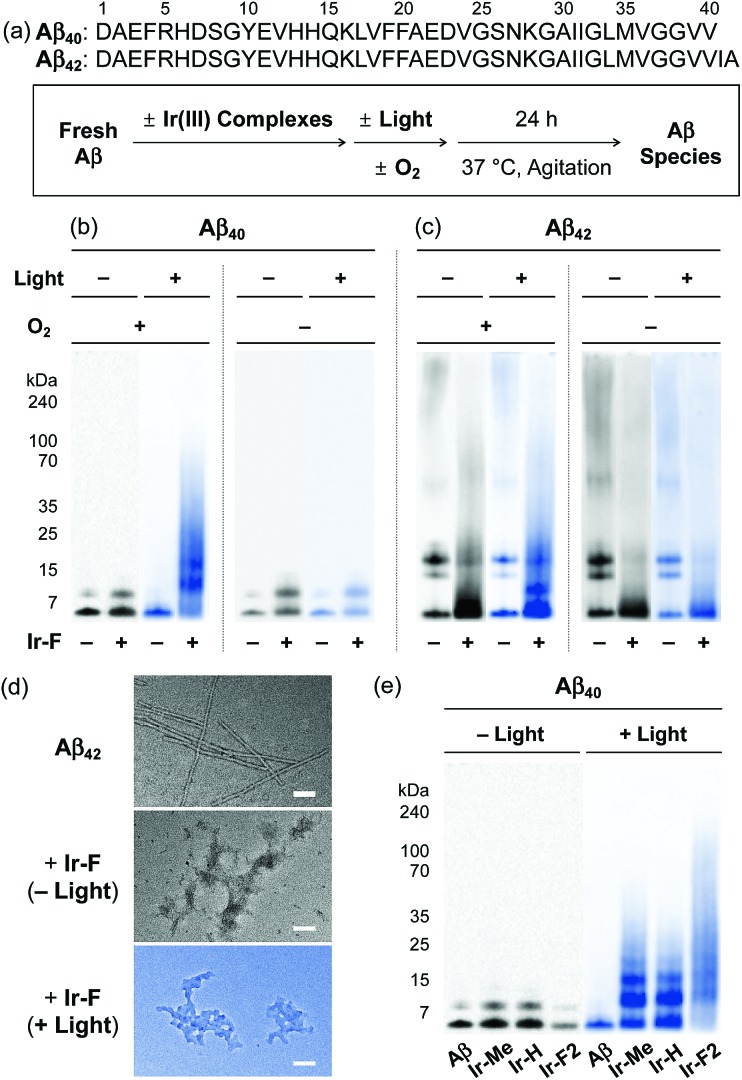
Change in the formation of Aβ aggregates by incubation with Ir(iii) complexes. (a) Sequences of Aβ_40_ and Aβ_42_ and scheme of the inhibition experiments. (b, c, and e) Analysis of the resultant Aβ_40_ and Aβ_42_ species generated under various conditions by gel/Western blot with an anti-Aβ antibody (6E10). (d) TEM images of the resultant Aβ_42_ aggregates (scale bar = 100 nm). Conditions: [Aβ] = 25 μM; [Ir(iii) complexes] = 250 μM; 37 °C; 24 h; constant agitation; 1 sun light for 10 min (for the samples treated with light).

Based on the photoirradiation-dependent Aβ modifications by Ir(iii) complexes, the impact of such variations on the aggregation of Aβ was determined employing Aβ_40_ and Aβ_42_, two main Aβ isoforms found in the AD-affected brain.^2^–^4^,^58^–^62^ For these experiments, freshly prepared Aβ solutions were treated with Ir(iii) complexes with and without light under both aerobic and anaerobic conditions. The molecular weight (MW) distribution and the morphology of resultant Aβ species were analyzed by gel electrophoresis with Western blotting (gel/Western blot) using an anti-Aβ antibody (6E10) and transmission electron microscopy (TEM), respectively (Fig. 3a).

Under aerobic conditions (Fig. 3b, left), the aggregation of Aβ_40_ was affected by treatment with **Ir-F** prompting a shift in the MW distributions in the absence of light. Photoactivation of the **Ir-F**-treated Aβ_40_ sample resulted in a more diverse MW distribution compared to that of the corresponding sample without light (light, MW ≤ 100 kDa; no light, MW < 15 kDa). The distinct modulation of Aβ_40_ aggregation upon addition of **Ir-F** with photoirradiation is likely a consequence of the complex's ability to generate ^1^O_2_ and oxidize Aβ through photoactivation as observed in our spectrometric studies (*vide supra*; Fig. 2). Therefore, the same experiments were performed under anaerobic conditions to directly monitor the role of O_2_ in **Ir-F′**s modulative reactivity against Aβ_40_ aggregation. In the absence of O_2_ (Fig. 3b, right), Aβ_40_ aggregation was also altered by **Ir-F** regardless of light treatment. Our results suggest that both light and O_2_ are important in the regulation of Aβ_40_ aggregation through coordination-/photo-mediated peptide oxidation triggered by **Ir-F**. In addition, in the absence of light and O_2_, Aβ_40_ aggregation is directed by the covalent interactions between **Ir-F** and the peptide. Similar modulation of Aβ_42_ aggregation was observed upon incubation with **Ir-F** exhibiting different MW distributions compared to the Aβ_42_ samples without **Ir-F** in the absence and presence of light and O_2_ (Fig. 3c). Moreover, smaller amorphous aggregates of both Aβ_40_ and Aβ_42_, reported to be less toxic,^63^,^64^ were visualized by TEM from the samples containing **Ir-F** regardless of irradiation (Fig. 3d and S12c†).

Furthermore, preformed Aβ aggregates, generated at various preincubation time points (*i.e.*, 2, 4, and 24 h), were disassembled and their aggregation pathways were altered when **Ir-F** was introduced (Fig. S13†). Such **Ir-F**-induced effects on preformed Aβ aggregates were observed to be dependent on photoirradiation. Moreover, the aggregation of both Aβ_40_ and Aβ_42_ was also changed with addition of the other Ir(iii) complexes (*i.e.*, **Ir-Me**, **Ir-H**, and **Ir-F2**) with and without light (Fig. 3e, S12 and S13†). In addition to Aβ, **Ir-F** was able to interact with and modify other amyloidogenic peptides [*i.e.*, α-synuclein (α-Syn) and human islet amyloid polypeptide (hIAPP)] affecting their aggregation pathways (Fig. S14†).

### Cytotoxicity of Aβ species generated upon incubation with Ir(iii) complexes

**Fig. 4:**
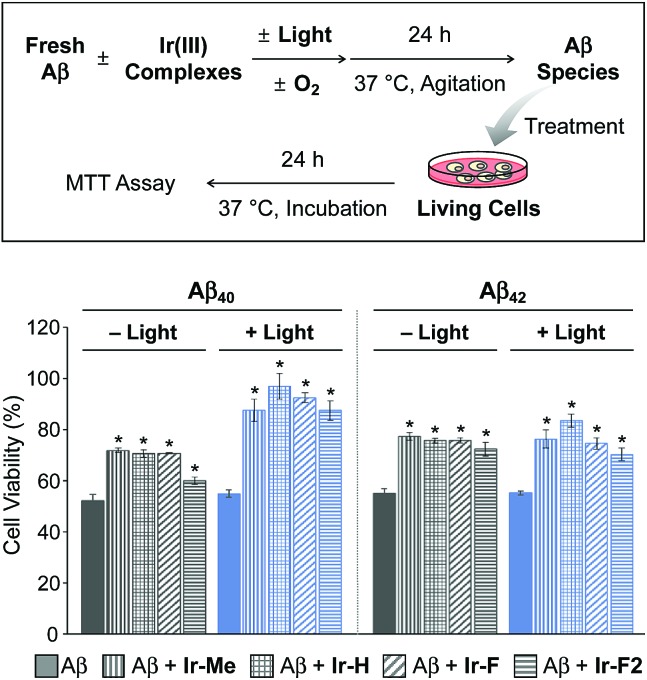
Viability of N2a cells upon 24 h treatment with Aβ species produced by incubation with Ir(iii) complexes for 24 h with and without light activation. Cell viability (%), measured by the MTT assay, was calculated and compared with that of samples treated with an equivalent amount of DMSO only. Conditions (final concentration): [Aβ] = 20 μM; [Ir(iii) complexes] = 5 μM. Error bars represent the standard error of the mean from three independent experiments. *P < 0.05.

Prior to cytotoxicity measurements, the resultant species upon 24 h treatment of Aβ_40_ with **Ir-F** with light exposure were incubated with murine Neuro-2a (N2a) neuroblastoma cells in order to determine their cellular uptake. As depicted in Fig. S15,† the lysates of the cells added with the resultant species for 24 h, analyzed by inductively coupled plasma-mass spectrometry (ICP-MS), indicated an Ir concentration of 39 μg L^–1^, demonstrating the cellular uptake of the species containing Ir(iii). Note that the Ir concentration (0.17 and 34 μg L^–1^) was measured from the lysates of the cells treated only with either Aβ_40_ or **Ir-F**, respectively. Moving forward, the toxicity of Aβ species produced by treatment with our Ir(iii) complexes was monitored by the MTT assay [MTT = 3-(4,5-dimethyl-2-thiazolyl)-2,5-diphenyl-2*H*-tetrazolium bromide] (Fig. 4). The cytotoxicity of Aβ_40_ species incubated with our Ir(iii) complexes was noticeably reduced in a photoirradiation-dependent manner. In the absence of light, the Aβ_40_ samples incubated with our Ir(iii) complexes exhibited a decrease in cytotoxicity (*ca.* 20%) compared to the sample of the complex-free Aβ_40_. As for the photoirradiated samples, Aβ_40_-induced toxicity was lowered by *ca.* 35% by treatment with our Ir(iii) complexes. This result suggests that modification of Aβ, such as oxidation, could attenuate Aβ-triggered toxicity in living cells.^65^ Furthermore, the cytotoxicity of Aβ_42_ species formed with Ir(iii) complexes was also diminished by *ca.* 20% regardless of photoactivation. Note that the survival (≥80%) of cells treated with our Ir(iii) complexes at the concentration used for cell studies with Aβ peptides was observed with and without light exposure (Fig. S16†).

### Ternary complexation with Aβ and intramolecular and intermolecular Aβ oxidation

**Fig. 5:**
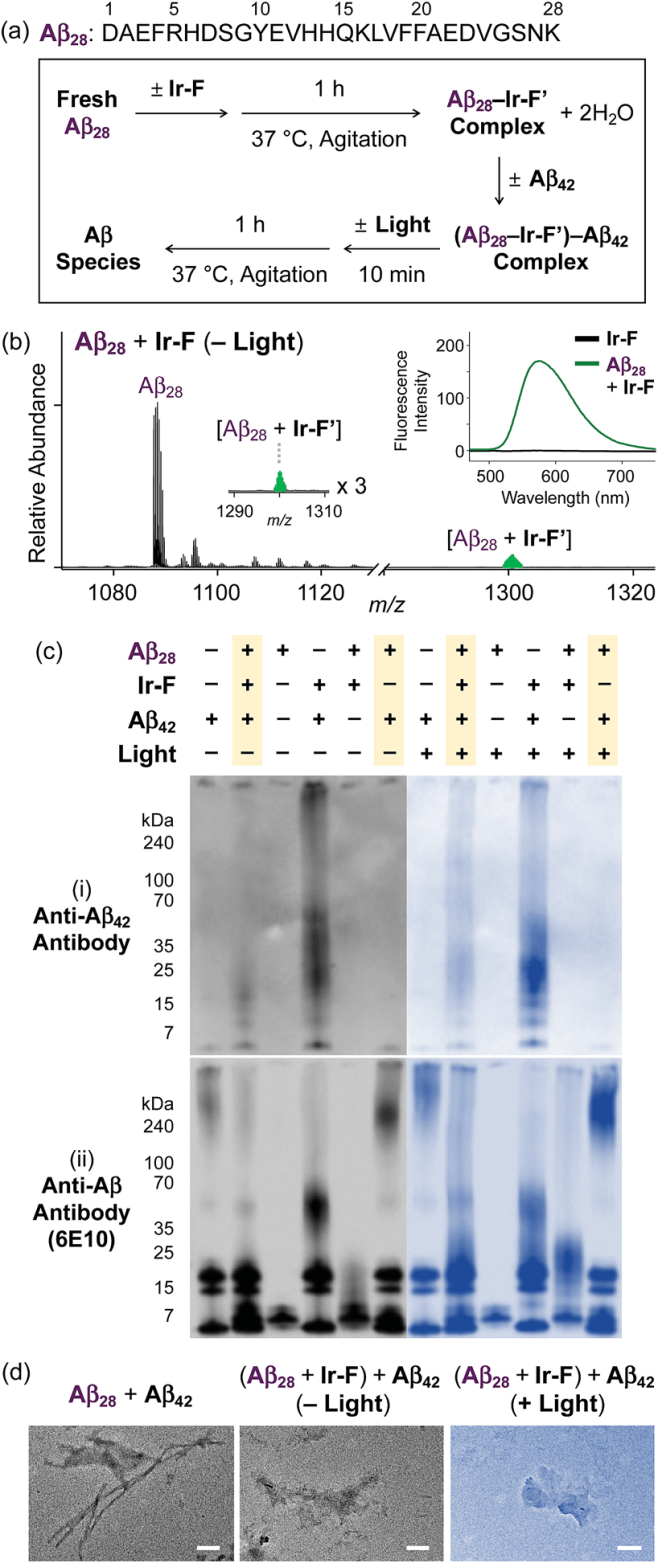
Impact of **Ir-F**-preincubated Aβ_28_ on the aggregation of Aβ_42_. (a) Sequence of Aβ_28_ and scheme of the experiments. (b) ESI-MS spectrum of +3-charged Aβ_28_ upon incubation with **Ir-F**. The complex peak is indicated in green. (Inset) Fluorescence response of **Ir-F** to Aβ_28_ (*λ*_ex_ = 433 nm). Charges are omitted in the MS spectra. Conditions: [Aβ_28_] = 100 μM; [**Ir-F**] = 100 μM; 37 °C; 1 h; no agitation; 1 sun light for 10 min (for the samples treated with light); aerobic conditions. (c) Analysis of the resultant Aβ species, obtained by addition of Aβ_42_ into **Ir-F** preincubated with Aβ_28_, by gel/Western blot with (i) anti-Aβ_42_ and (ii) anti-Aβ (6E10) antibodies. (d) TEM images of the resultant Aβ aggregates (scale bar = 100 nm). Conditions: [Aβ_28_] = 50 μM; [**Ir-F**] = 10 μM; [Aβ_42_] = 20 μM; 37 °C; 2 h; constant agitation; 1 sun light for 10 min (for the samples treated with light); aerobic conditions.

**Fig. 6:**
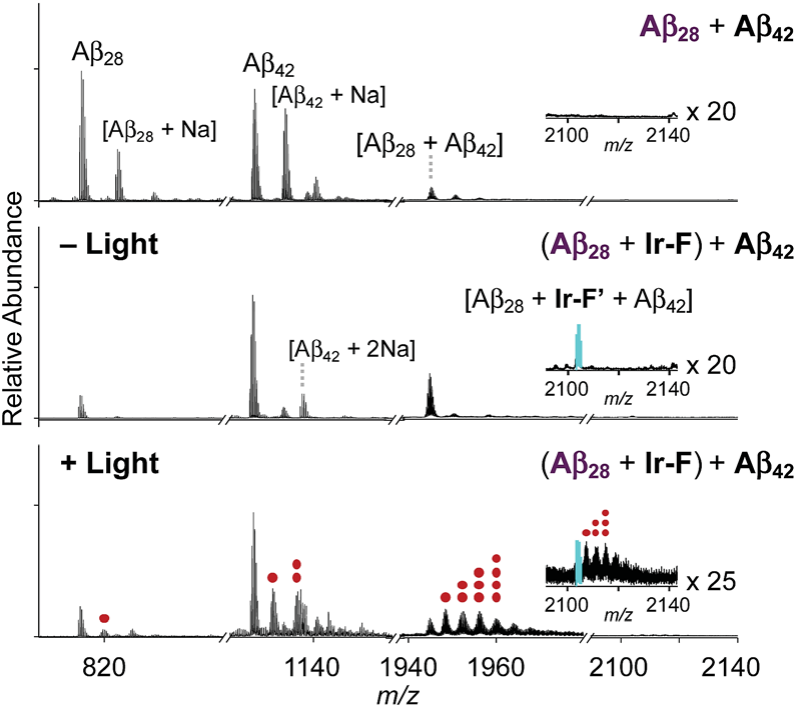
ESI-MS spectra of **Ir-F**-incubated +4-charged Aβ_28_ and Aβ_42_ with and without light. The peak indicated in cyan refers to a ternary complex of Aβ_28_, **Ir-F′**, and Aβ_42_. The peaks corresponding to oxidized peptides (*i.e.*, Aβ_28_, Aβ_42_, and Aβ_28_ with Aβ_42_) are indicated with red dots. The number of red dots represents the number of oxygen atoms incorporated into each peptide. Charges are omitted in the MS spectra. Conditions: [Aβ_28_] = 100 μM; [**Ir-F**] = 100 μM; [Aβ_42_] = 100 μM; 37 °C; 2 h; no agitation; 1 sun light for 10 min (for the samples treated with light); aerobic conditions.

Premised on **Ir-F′**s covalent bond formation with Aβ and oxidation of Aβ (*vide supra*), additional studies regarding ternary complexation and promotion of intermolecular oxidation of Aβ were carried out employing **Ir-F** (Fig. 5). Aβ_28_, a fragment of Aβ equipped with the metal binding and self-recognition sites of the peptide with a relatively low propensity to aggregate than the full-length peptides, Aβ_40_ and Aβ_42_,^1^,^66^–^68^ was used to form a complex with **Ir-F′** (Fig. 2b) as evidenced by ESI-MS (1301 *m*/*z*; Fig. 5b) and increased fluorescence (Fig. 5b, inset). As shown in Fig. 5a, following incubation, the sample of the Aβ_28_–**Ir-F′** complex was treated with freshly prepared Aβ_42_ to monitor its effect on Aβ_42_ aggregation. Based on the gel/Western blot and TEM analyses, the aggregation of Aβ_42_ was modulated by the Aβ_28_–**Ir-F′** complex (Fig. 5c and d). Such modulative reactivity of the Aβ_28_–**Ir-F′** complex was also observed against Aβ_40_ aggregation (Fig. S17†). Our mass spectrometric studies confirmed that such control of Aβ_42_ aggregation by the Aβ_28_–**Ir-F′** complex was a result of ternary complex formation with Aβ_42_, *i.e.*, (Aβ_28_–**Ir-F′**)–Aβ_42_, and (ii) oxidation of Aβ, both intramolecular and intermolecular, upon photoactivation (Fig. 6). Based on previous reports detailing intermolecular interactions between Aβ peptides, hydrophobic interactions between the self-recognition sites (LVFFA; Fig. 3a and 5a) of Aβ are likely responsible for ternary complexation,^1^,^2^,^69^ consequentially altering the aggregation pathways of Aβ in the absence of photoirradiation. Furthermore, these studies indicate that intermolecular oxidation of Aβ can be promoted by **Ir-F** upon photoactivation (Fig. 6, S18, and S19†). This observation may explain the distinct difference between the modulation of Aβ aggregation with and without light as the intermolecular oxidation of Aβ by Ir(iii) complexes could modify Aβ at sub-stoichiometric levels.

## Conclusions

Effective chemical strategies (*i.e.*, coordination to Aβ and coordination-/photo-mediated oxidation of Aβ) for modification of Aβ peptides using a single Ir(iii) complex were rationally developed. Such dual mechanisms (*i.e.*, coordination and oxidation) exhibiting photo-dependency for altering Aβ peptides are novel and effective in controlling peptide aggregation and cytotoxicity. Our Ir(iii) complexes can covalently bind to Aβ by replacing two H_2_O molecules bound to the Ir(iii) center with Aβ regardless of light and O_2_ [coordination to Aβ; Fig. 1a(i)]. In the presence of light and O_2_, Ir(iii) complexes bound to Aβ are capable of inducing the intramolecular and intermolecular oxidation of Aβ at His13, His14, and/or Met35 [oxidation of Aβ; Fig. 1a(ii)]. Taken together, our multidisciplinary studies demonstrate the feasibility of establishing new chemical approaches towards modification of amyloidogenic peptides (*e.g.*, Aβ) using transition metal complexes designed based on their coordination and photophysical properties. In general, chemical modifications in peptides of interest can assist in furthering our understanding of principles of their properties, such as peptide assembly. Furthermore, peptide aggregation and cytotoxicity can be affected by biomolecules, including lipid membranes;^70^–^73^ thus, the regulatory reactivity of Ir(iii) complexes towards amyloidogenic peptides in the presence of lipid membranes will be investigated in the future.

## Conflicts of interest

There are no conflicts to declare.

## Supplementary Material

Supplementary information
