# Supplementary material for: Chemical strategies to modify amyloidogenic peptides using iridium(iii) complexes: coordination and photo-induced oxidation
Source: Chem Sci. 2019 Jun 5;10(28):6855–62. doi: 10.1039/c9sc00931k (PMC6657414; doi:10.1039/c9sc00931k)
Supplement: Supplementary file 1 [file SC-010-C9SC00931K-s001.pdf]

Electronic Supplementary Material (ESI) for Chemical Science.
This journal is © The Royal Society of Chemistry 2019

Supplementary Information

for

Chemical strategies to modify amyloidogenic peptides by iridium(III) complexes: Coordination and photo-induced oxidation

Juhye Kang,[‡ab] Jung Seung Nam,[‡b] Hyuck Jin Lee,[ac] Geewoo Nam,[ab] Hyun-Woo Rhee,[d] Tae-Hyuk Kwon,*[b] and Mi Hee Lim*[a]

[a]Department of Chemistry, Korea Advanced Institute of Science and Technology (KAIST), Daejeon 34141, Republic of Korea

[b]Department of Chemistry, Ulsan National Institute of Science and Technology (UNIST), Ulsan 44919, Republic of Korea

[c]Department of Chemistry Education, Kongju National University, Gongju 32588, Republic of Korea

[d]Department of Chemistry, Seoul National University, Seoul 08826, Republic of Korea

*To whom correspondence should be addressed: miheelim@kaist.ac.kr and kwon90@unist.ac.kr

[‡]These authors contributed equally to this work.

Table of Contents

Experimental Section S4

Materials and Methods S4

Synthesis of Ir(III) Complexes S5

Photophysical Properties of Ir(III) Complexes S10

Binding Affinities of Ir(III) Complexes S11

Singlet Oxygen ($^1O_2$) Generation by Ir(III) Complexes S12

Superoxide Anion Radical ($O_2^{\bullet-}$) Production by Ir(III) Complexes S12

Dot Blot Assay S12

Electrospray Ionization-Ion Mobility-Mass Spectrometry (ESI-IM-MS) S13

Aβ Aggregation Experiments S14

Gel Electrophoresis with Western Blotting (Gel/Western Blot) S15

Transmission Electron Microscopy (TEM) S15

Inductively Coupled Plasma-Mass Spectrometry (ICP-MS) S16

Cytotoxicity Studies S16

Fig. S1 ^1H and ^{13}C NMR spectra of **Ir-Me** S18

Fig. S2 ^1H, ^{13}C, and ^{19}F NMR spectra of **Ir-F** S19

Fig. S3 ^1H, ^{13}C, and ^{19}F NMR spectra of **Ir-F2** S20

Fig. S4 Fluorescence responses of Ir(III) complexes to His and Aβ in DMSO S21

Fig. S5 His binding of Ir(III) complexes S22

Fig. S6 Analysis of the amount of singlet oxygen ($^1O_2$) generated by Ir(III) complexes in the absence and presence of His S23

**Fig. S7**	Binding affinities of **Ir-F** towards different Aβ species	S25
**Fig. S8**	Determination of the amount of superoxide anion radicals ($O_2^{\bullet-}$) produced by Ir(III) complexes with and without His	S26
**Fig. S9**	ESI-MS spectra of +3-charged $A\beta_{40}$ treated with **Ir-Me**, **Ir-H**, and **Ir-F2** in the absence and presence of light	S27
**Fig. S10**	IM-MS spectra of +3-charged $A\beta_{40}$ incubated with and without Ir(III) complexes	S28
**Fig. S11**	Analysis of oxidized Aβ species produced by photoactivated **Ir-F**	S29
**Fig. S12**	Influence of Ir(III) complexes on the formation of Aβ aggregates	S31
**Fig. S13**	Impact of Ir(III) complexes on the disassembly of preformed $A\beta_{42}$ aggregates produced at various incubation time points	S32
**Fig. S14**	Ability of **Ir-F** to modify other amyloidogenic peptides, *i.e.*, α-Syn and hIAPP, and control their aggregation pathways	S33
**Fig. S15**	Cellular uptake of Aβ species generated by light-activated **Ir-F**	S35
**Fig. S16**	Viability of N2a cells with Ir(III) complexes	S36
**Fig. S17**	Impact of **Ir-F**-preincubated $A\beta_{28}$ on $A\beta_{40}$ aggregation	S37
**Fig. S18**	ESI-MS spectra of +3-charged $A\beta_{28}$ incubated with **Ir-F** in the absence and presence of light	S38
**Fig. S19**	ESI-MS spectra of +3-charged $A\beta_{42}$ treated with **Ir-F** in the absence and presence of light	S39
**References**		S40

Experimental Section

Materials and Methods. All reagents were purchased from commercial suppliers and used as received unless otherwise noted. Analyses of small molecules by nuclear magnetic resonance (NMR) spectroscopy, Fourier-transform infrared (FT-IR) spectroscopy, and mass spectrometry (MS) were conducted on a Bruker AVANCE III HD NMR spectrometer, Varian Cary 620/670 FT-IR spectrometer (UNIST Central Research Facilities, Ulsan, Republic of Korea), and a Thermo Scientific Q Exactive Hybrid Quadrupole-Orbitrap Mass Spectrometer, respectively. $A\beta_{40}$, $A\beta_{42}$, $A\beta_{16}$, and $A\beta_{28}$ were purchased from Anygen ($A\beta_{40}$ = DAEFRHDSGYEVHHQKLVFFAEDVGS-NKGAIIGLMVGGVV; $A\beta_{42}$ = DAEFRHDSGYEVHHQKLVFFAEDVGSNKGAIIGLMVGG-VVIA; Nam-myun, Jangseong-gun, Republic of Korea) and AnaSpec ($A\beta_{16}$ = DAEFRHDSGYEVHHQK; $A\beta_{28}$ = DAEFRHDSGYEVHHQKLVFFAEDVGSNK; Fremont, CA, USA), respectively. α-Synuclein (α-Syn) and human islet amyloid polypeptide (hIAPP) were obtained from Anaspec and Peptron, respectively [α-Syn = MDVFMKGLSKAKEGVVAAAEK-TKQGVAEAAGKTKEGVLYVGSKTKEGVVHGVATVAEKTKEQVTNVGGAVVTGVTAV AQKTVEGAGSIAAATGFVKKDQLGKNEEGAPQEGILEDMPVDPDNEAYEMPSEEGYQD YEPEA (Fremont, CA, USA); hIAPP = KCNTATCATQRLANFLVHSSNNFGAILSSTNVGSN-KCNTATCATQRLANFLVHSSNNFGAILSSTNVGSNTY-NH_2 (Daejeon, Republic of Korea)]. Double distilled H_2O was obtained from a Milli-Q Direct 16 system (Merck KGaA, Darmstadt, Germany). 1 sun light (100 mW cm^{-2}) was treated from a Newport IQE-200 solar simulator (Irvine, CA, USA). Anaerobic reactions were performed in a N_2-filled glovebox (Korea Kiyon, Bucheon-si, Gyeonggi-do, Republic of Korea). Transmission electron microscopical images were taken by a JEOL JEM-2100 transmission electron microscope (UNIST Central Research Facilities, Ulsan, Republic of Korea) and a Tecnai F30 (FEI) transmission electron microscope [KAIST Analysis

Center for Research Advancement (KARA), Daejeon, Republic of Korea]. Analysis by electrospray ionization-ion mobility-mass spectrometry (ESI-IM-MS) was carried out using a Waters Synapt G2-Si quadrupole time-of-flight ion mobility mass spectrometer (DGIST Center for Core Research Facilities, Daegu, Republic of Korea) and an Agilent 6530 Accurate-Mass Q-TOF LC/MS equipped with an ESI source. Photophysical properties were measured by a Shimadzu UV-2600 UV-visible (UV-Vis) spectrophotometer and a Varian Cary Eclipse fluorescence spectrophotometer (UNIST Central Research Facilities, Ulsan, Republic of Korea). Time-correlated single photon counting (TCSPC) was conducted for lifetime measurements with Ti:sapphire laser Mira900 (Coherent, Santa Clara, CA, USA), monochromator Acton Series SP-2150i (Princeton Instruments, Acton, MA, USA), and TCSPC module PicoHarp 300 (PicoQuant, Berlin, Germany), with micro channel plate photomultiplier tube (MCP-PMT) R3809U-59 (Hamamatsu, Shizuika-ken, Japan) and fitted by PicoQuant FluoFit software (UNIST Central Research Facilities, Ulsan, Republic of Korea). Agilent inductively coupled plasma-mass spectrometry (ICP-MS) 7700S [KAIST Analysis Center for Research Advancement (KARA), Daejeon, Republic of Korea] was employed to detect an Ir concentration in cell lysates. A SpectraMax M5e microplate reader (Molecular Devices, Sunnyvale, CA, USA) was used to measure the absorbance and emission for the MTT assay [MTT = 3-(4,5-dimethyl-2-thiazolyl)-2,5-diphenyl-2*H*-tetrazolium bromide, Sigma-Aldrich, St. Louis, MO, USA] and the DHR123 assay (DHR123 = dihydrorhodamine 123; Thermo Scientific, MA, USA), respectively.

Synthesis of Ir(III) Complexes

Preparation of 2-Me. 2-Me was synthesized by modifications of previously reported procedures.[1] $CH_3(CH_2)_3Li$ (*n*-BuLi; 2.5 M; 7.0 mL, 18 mmol) was slowly dropped to a solution of 4-

bromotoluene (**1-Me**; 2.0 g, 12 mmol) in tetrahydrofuran (THF) under -78 °C, and the solution was vigorously stirred for 1 h. $B(OCH_3)_3$ (2.2 g, 21 mmol) was slowly added to the solution, and the reaction mixture was stirred for additional 4 h. The reaction mixture was quenched with H_2O. The product was extracted with CH_2Cl_2 three times. The collected organic solution was treated with $MgSO_4$ and removed under the reduced pressure to obtain a white powder product. (1.2 g, yield 64%). ^{1}H NMR [400 MHz, $CDCl_3$, δ (ppm)]: 8.13 (d, J = 7.9 Hz, 2H), 7.31 (d, J = 7.5 Hz, 2H), 2.45 (s, 3H).

Preparation of 2-F. 2-F was synthesized by the same procedure as **2-Me** using 4-fluoroiodobenzene (**1-F**; 3.0 g, 14 mmol), *n*-BuLi (2.5 M; 8.1 mL, 20 mmol), and $B(OCH_3)_3$ (2.5 g, 24 mmol) (white powder; 820 mg, yield 43%).[1] ^{1}H NMR [400 MHz, $CDCl_3$, δ (ppm)]: 8.19-8.24 (m, 2H), 7.19 (t, J = 8.8 Hz, 2H).

Preparation of 3-H. 3-H was synthesized by modifications of previously reported procedures.[2] 2-Chloroquinoline (400 mg, 2.4 mmol), phenylboronic acid (**2-H**; 358 mg, 2.9 mmol), and $Pd(PPh_3)_4$ (141 mg, 0.12 mmol) were added to a mixture of degassed THF and K_2CO_3 (2 M, aq) (1:1). The solution was refluxed under N_2 (g) overnight. After cooling down to room temperature, the solution was concentrated, and H_2O was added into the resulting solution. The organic phase was extracted with ethyl acetate (EtOAc) three times. The collected organic solution was treated with $MgSO_4$ and removed under the reduced pressure. The crude materials were purified by column chromatography (SiO_2, 10:1 hexanes:EtOAc, R_f = 0.6; white powder; 297 mg, yield 59%). ^{1}H NMR [400 MHz, DMSO-d_6, δ (ppm)]: 8.45 (d, J = 8.8 Hz, 1H), 8.26 (d, J = 7.2 Hz, 2H), 8.14 (d, J = 8.0 Hz, 1H), 8.06 (d, J = 8.0 Hz, 1H), 7.98 (d, J = 8.0 Hz, 1H), 7.77 (t, J = 7.2 Hz, 1H), 7.46-

7.62 (m, 4H).

Preparation of 3-Me. 3-Me was synthesized by the same procedure as **3-H** using 2-chloroquinoline (600 mg, 3.7 mmol), **2-Me** (600 mg, 4.4 mmol), and $Pd(PPh_3)_4$ (212 mg, 0.18 mmol) (10:1 hexanes:EtOAc, R_f = 0.6; white powder; 733 mg, yield 91%). ^{1}H NMR [400 MHz, DMSO-d_6, δ (ppm)]: 8.18 (t, J = 9.2 Hz, 2H), 8.08 (d, J = 8.0 Hz, 2H), 7.86 (d, J = 8.4 Hz, 1H), 7.82 (dd, J = 8.1, 1.5 Hz, 1H), 7.72 (ddd, J = 8.4, 6.9, 1.5 Hz, 1H), 7.52 (ddd, J = 8.1, 6.8, 1.2 Hz, 1H), 7.34 (d, J = 8.1 Hz, 2H), 2.44 (s, 3H). ^{13}C NMR [100 MHz, DMSO-d_6, δ (ppm)]: 157.3, 148.3, 139.4, 136.9, 136.7, 129.7, 129.6, 129.4, 127.5, 127.1, 126.8, 126.1, 118.9, 21.4. FT-IR (neat cm^{-1}): 3055, 3026, 2958, 2914, 2858, 1597, 1552, 1496, 1431, 1373, 1319, 1288, 1244, 1213, 1184, 1157, 1126, 1111, 1053, 1016, 974, 949, 814, 789, 746, 717. HRMS for $[C_{16}H_{13}N + H]^+$ Calcd, 220.1121; found, 220.1116.

Preparation of 3-F. 3-F was synthesized by the same procedure as **3-H** using 2-chloroquinoline (500 mg, 3.1 mmol), **2-F** (500 mg, 3.6 mmol), and $Pd(PPh_3)_4$ (176 mg, 0.15 mmol) (10:1 hexanes:EtOAc, R_f = 0.7; white powder; 616 mg, yield 90%). ^{1}H NMR [400 MHz, DMSO-d_6, δ (ppm)]: 8.22 (d, J = 8.4 Hz, 1H), 8.17 (t, J = 8.0 Hz, 3H), 7.83 (dd, J = 8.8 Hz, 2H), 7.73 (ddd, J = 8.5, 7.0, 1.5 Hz, 1H), 7.53 (ddd, J = 8.1, 6.8, 1.2 Hz, 1H), 7.21 (t, J = 8.8 Hz, 2H). ^{13}C NMR [100 MHz, DMSO-d_6, δ (ppm)]: 168.8, 162.9, 160.4, 148.2, 143.8, 143.8, 143.3, 140.9, 131.7, 129.1, 129.0, 128.9, 127.7, 127.1, 125.9, 119.7, 119.6, 117.5, 109.5, 109.3. FT-IR (neat cm^{-1}): 3067, 1736, 1594, 1558, 1515, 1495, 1432, 1324, 1286, 1270, 1230, 1162, 1125, 1098, 1047, 1012, 977, 940, 852, 820, 789, 758, 710, 691, 673. HRMS for $[C_{15}H_{10}FN + H]^+$ Calcd, 224.0870; found, 224.0864.

Preparation of 3-F2. 3-F2 was synthesized by the same procedure as **3-H** using 2-chloroquinoline (860 mg, 5.3 mmol), 2,4-difluorophenylboronic acid (**2-F2**; 996 mg, 6.3 mmol), and $Pd(PPh_3)_4$ (304 mg, 0.26 mmol) (10:1 hexanes:EtOAc, $R_f = 0.7$; white powder; 742 mg, yield 59%). ^{1}H NMR [400 MHz, DMSO-d_6, δ (ppm)]: 8.46 (d, J = 8.8 Hz, 1H), 8.07-8.14 (m, 2H), 8.02 (d, J = 8.0 Hz, 1H), 7.88 (dd, J = 8.4, 2.4 Hz, 1H), 7.80 (t, J = 7.6 Hz, 1H), 7.63 (t, J = 7.6 Hz, 1H), 7.43 (t, J = 9.2 Hz, 1H), 7.28 (t, J = 8.8 Hz, 1H). ^{13}C NMR [100 MHz, DMSO-d_6, δ (ppm)]: 164.5, 162.0, 159.5, 152.8, 148.0, 137.3, 133.2, 130.5, 129.5, 128.3, 127.3, 124.4, 122.3, 112.7, 105.1. FT-IR (neat cm^{-1}): 3057, 2954, 2923, 2853, 1676, 1613, 1596, 1497, 1456, 1430, 1418, 1318, 1294, 1264, 1134, 1096, 1053, 970, 942, 836, 808, 758. HRMS for $[C_{15}H_9F_2N + H]^+$ Calcd, 242.0776; found, 242.0772.

Preparation of Ir-H. Ir-H was synthesized by modifications of previously reported methods.[3,4] **3-H** (859 mg, 4.2 mmol) was added to a solution of $IrCl_3 \cdot nH_2O$ (500 mg, 1.7 mmol) in a mixture of 2-methoxyethanol and H_2O (3:1). The solution was refluxed under N_2 (g) for 24 h. After cooling down to room temperature, brown precipitates were obtained by addition of H_2O. The crude product (the cyclometalated chloride-bridged Ir(III) dimer, [Ir(μ-Cl)(**3-H**)$_2$]$_2$) was washed with hexanes and cold diethyl ether (Et_2O) several times, dried, and used without further purification.[5]

A solution of [Ir(μ-Cl)(**3-H**)$_2$]$_2$ (150 mg, 0.12 mmol) and AgOTf (91 mg, 0.35 mmol) was dissolved in a mixture of CH_2Cl_2 and CH_3OH (1:1). The solution was stirred under inert conditions for 6 h. The reaction mixture was filtered through Celite to remove precipitates. The filtrate was concentrated in vacuo, and the resultant residues were solidified with hexanes. The target product (orange powder; 41 mg, yield 22%) was collected by additional crystallization with $CHCl_3$ and

hexanes. ^{1}H NMR [400 MHz, DMSO-d_6, δ (ppm)]: 9.12 (d, J = 8.8 Hz, 2H), 8.68 (d, J = 8.8 Hz, 2H), 8.46 (d, J = 8.8 Hz, 2H), 8.16 (d, J = 8.0 Hz, 2H), 7.96 (d, J = 7.6 Hz, 2H), 7.87 (t, J = 8.4 Hz, 2H), 7.73 (t, J = 7.2 Hz, 2H), 6.86 (t, J = 7.2 Hz, 2H), 6.55 (t, J = 7.2 Hz, 2H), 6.03 (d, J = 7.6 Hz, 2H).

Preparation of Ir-Me. Ir-Me was synthesized by the same procedure as **Ir-H** using **3-Me** (700 mg, 3.2 mmol), $IrCl_3 \cdot nH_2O$ (433 mg, 1.5 mmol), [Ir(μ-Cl)(**3-Me**)$_2$]$_2$ (132 mg, 0.10 mmol), and AgOTf (77 mg, 0.30 mmol) (red powder; 72 mg, yield 44%). ^{1}H NMR [400 MHz, DMSO-d_6, δ (ppm)]: 9.06 (d, J = 8.4 Hz, 2H), 8.66 (d, J = 8.8 Hz, 2H), 8.41 (d, J = 8.8 Hz, 2H), 8.16 (dd, J = 8.1, 1.5 Hz, 2H), 7.83-7.90 (m, 4H), 7.72 (ddd, J = 8.0, 6.8, 1.1 Hz, 2H), 6.72 (d, J = 8.8 Hz, 2H), 5.89 (s, 2H), 1.85 (s, 6H). ^{13}C NMR [100 MHz, DMSO-d_6, δ (ppm)]: 169.8, 148.4, 144.0, 141.5, 140.2, 139.1, 134.8, 131.2, 128.7, 127.5, 126.6, 126.5, 126.3, 123.0, 122.3, 119.1, 117.1, 21.2. FT-IR (neat cm^{-1}): 3361 (br), 3088, 2916, 1606, 1579, 1554, 1520, 1451, 1430, 1338, 1247, 1226, 1170, 1075, 1027, 988, 962, 876, 817, 785, 762, 734, 681, 662. HRMS for $[M - 2H_2O - OTf]^+$ Calcd, 629.1563; found, 629.1564.

Preparation of Ir-F. Ir-F was synthesized by the same procedure as **Ir-H** using **3-F** (616 mg, 2.8 mmol), $IrCl_3 \cdot nH_2O$ (358 mg, 1.2 mmol), [Ir(μ-Cl)(**3-F**)$_2$]$_2$ (134 mg, 0.10 mmol), and AgOTf (77 mg, 0.30 mmol) (orange powder; 67 mg, yield 41%). ^{1}H NMR [400 MHz, DMSO-d_6, δ (ppm)]: 9.08 (d, J = 8.8 Hz, 2H), 8.75 (d, J = 8.7 Hz, 2H), 8.50 (d, J = 8.8 Hz, 2H), 8.21 (dd, J = 8.1, 1.5 Hz, 2H), 8.12 (dd, J = 8.7, 5.9 Hz, 2H), 7.92 (ddd, J = 8.7, 6.8, 1.5 Hz, 2H), 7.78 (ddd, J = 8.0, 6.8, 1.1 Hz, 2H), 6.79 (td, J = 8.8, 2.6 Hz, 2H), 5.67 (dd, J = 9.9, 2.5 Hz, 2H). ^{13}C NMR [100 MHz, DMSO-d_6, δ (ppm)]: 168.8, 162.9, 160.4, 148.2, 143.8, 143.8, 143.3, 140.9, 131.7, 129.1, 129.0,

128.9, 127.7, 127.1, 125.9, 122.3, 119.7, 119.6, 119.1, 117.5, 109.5, 109.3. ^{19}F NMR [376 MHz, DMSO-d_6, δ(ppm)]: -77.75 (s), -109.34 (m). FT-IR (neat cm^{-1}): 3359 (br), 3088, 2933, 2860, 1595, 1571, 1550, 1516, 1469, 1451, 1432, 1395, 1341, 1297, 1232, 1194, 1182, 1149, 1070, 1025, 980, 962, 889, 864, 847, 834, 804, 782, 744, 709, 675, 662. HRMS for [M - 2H_2O - OTf]$^+$ Calcd, 637.1062; found, 637.1060.

Preparation of Ir-F2. Ir-F2 was synthesized by the same procedure as **Ir-H** using **3-F2** (927 mg, 3.8 mmol), $IrCl_3 \cdot nH_2O$ (459 mg, 1.5 mmol), [Ir(μ-Cl)(**3-F2**)$_2$]$_2$ (100 mg, 0.071 mmol), and AgOTf (54 mg, 0.21 mmol) (orange powder; 18 mg, yield 15%). ^{1}H NMR [400 MHz, DMSO-d_6, δ(ppm)]: 8.89 (d, J = 8.8 Hz, 2H), 8.80 (d, J = 9.2 Hz, 2H), 8.51 (d, J = 9.2 Hz, 2H), 8.22 (d, J = 8.4 Hz, 2H), 7.92 (t, J = 7.2 Hz, 2H), 7.79 (t, J = 7.2 Hz, 2H), 6.86 (t, J = 11 Hz, 2H), 5.63 (d, J = 8.8 Hz, 2H). ^{13}C NMR [100 MHz, DMSO-d_6, δ(ppm)]: 167.0, 166.9, 163.4, 162.4, 160.8, 160.7, 159.9, 159.8, 148.5, 145.3, 142.2, 137.6, 132.5, 132.0, 131.9, 131.2, 130.7, 129.4, 128.4, 128.0, 127.9, 127.5, 126.6, 122.8, 120.4, 120.2, 119.6, 117.3, 117.1, 99.7, 99.4, 99.1. ^{19}F NMR [376 MHz, DMSO-d_6, δ(ppm)]: -77.75 (s), -106.58 (m, J = 11.3 Hz), -107.85 (m, J = 11.3 Hz). FT-IR (neat cm^{-1}): 3319 (br), 3081, 2935, 1597, 1556, 1518, 1433, 1411, 1317, 1295, 1216, 1190, 1162, 1106, 1019, 992, 830, 756. HRMS for [M - 2H_2O - OTf]$^+$ Calcd, 673.0868; found, 673.0864.

Photophysical Properties of Ir(III) Complexes. Absorption and emission spectra of the solution of (i) [Ir(III) complexes (**Ir-Me**, **Ir-H**, **Ir-F**, or **Ir-F2**; 20 μM in H_2O) with or without histidine (His) (100 μM)], (ii) [**Ir-F** (20 μM in H_2O) with or without $A\beta_{28}$ (100 μM)], (iii) [**Ir-Me**, **Ir-H**, **Ir-F**, or **Ir-F2** (20 μM in DMSO) with or without His or $A\beta_{16}$ (100 μM)], (iv) [**Ir-F** (250 μM in H_2O) with or without $A\beta_{40}$ (25 μM) in H_2O at 37 °C for 24 h with constant agitation upon 10 min

exposure to 1 sun light followed by dilution by *ca.* 11.5 fold with cell growth media or H_2O (*vide infra*)], and (v) [**Ir-F** (20 μM in H_2O) with α-Syn or hIAPP (100 μM)] were recorded at 298 K by UV-Vis and fluorescence spectroscopy, respectively. Quantum yield (Φ) was calculated following previously reported procedures. [Ir(2-phenylquinoline)$_2$(2,2'-bipyridine)]Cl (Φ_{ref} = 0.53 in H_2O, 1% v/v DMSO) was used as a reference.[6] For lifetime measurements, time-correlated single photon counting (TCSPC) was performed. The second harmonic generation (SHG = 420 nm) of a tunable Ti:sapphire laser with *ca.* 150 fs pulse width and 76 MHz repetition rate was used as an excitation source. The emission was spectrally resolved by using some collection optics and a monochromator. The TCSPC module with an MCP-PMT was used for ultrafast detection. The total instrument response function (IRF) for measuring the fluorescence decay was less than 150 ps, and the temporal time resolution was less than 10 ps. The deconvolution of the actual fluorescence decay and IRF was fitted by using the FluoFit software to deduce the time constant associated with each exponential decay. Radiative decay constant (k_r) and nonradiative decay constant (k_{nr}) were calculated on the basis of previously reported equations.[7]

Binding Affinities of Ir(III) Complexes. The solution of **Ir-Me**, **Ir-H**, **Ir-F**, or **Ir-F2** (20 μM in H_2O, 1% v/v DMSO) with or without His (200 μM) was measured by ESI-MS (Agilent 1200 and Bruker HCT). Dissociation constants (K_d) were determined through His and Aβ titration experiments. The fluorescence responses of **Ir-Me**, **Ir-H**, **Ir-F**, or **Ir-F2** (2 or 4 μM in H_2O; 1% v/v DMSO) to His (0-60 μM) or Aβ species [0-120 μM; monomers ($A\beta_{16}$; 0 h incubation), oligomers ($A\beta_{42}$; 2 h incubation), and fibrils ($A\beta_{42}$; 48 h incubation)] were measured at room temperature. The resultant titration points were fitted to the Hill's equation [$y = V_{max} \{x^n / (x^n + k^n)\}$] [$V_{max}$, emission intensity of complete saturation; n, cooperativity; k, half maximal effective

concentration (EC_{50})] in non-linear curve fit (OriginPro 2015, OriginLab Corporation, Northampton, MA, USA) and the K_d values were calculated by the obtained constant [$K_d = k^n$].

Singlet Oxygen ($^1O_2$) Generation by Ir(III) Complexes. The amounts of singlet oxygen ($^1O_2$) generated upon treatment with **Ir-Me**, **Ir-H**, **Ir-F**, and **Ir-F2** were determined according to the method reported previously.[6] Solutions containing Ir(III) complexes (10 μM in H_2O, 1% v/v DMSO) and 9,10-anthracenediyl-bis(methylene)dimalonic acid (ABDA as a $^1O_2$ substrate, 100 μM) with or without His (10 μM) were irradiated with 40% of 1 sun light (40 $mWcm^{-2}$; optimized intensity for ABDA not to be degraded). The absorbance of ABDA was measured for 7.5 min with a 2.5 min interval.

Superoxide Anion Radical ($O_2^{\bullet-}$) Production by Ir(III) Complexes. DHR123 was utilized for measuring the amount of superoxide anion radicals ($O_2^{\bullet-}$). The solution containing Ir(III) complexes (10 μM), DHR123 (10 μM), and His (100 μM) was irradiated with 1 sun light for 5 min. The fluorescence emission for the DHR123 assay was recorded onto a microplate reader (λ_{ex} = 485 nm).

Dot Blot Assay. Aβ species [5 μL (50 μM); monomers ($A\beta_{16}$; 0 h incubation), oligomers ($A\beta_{42}$; 2 h incubation), and fibrils ($A\beta_{42}$; 48 h incubation)] were spotted on a nitrocellulose membrane and the membrane was blocked with a bovine serum albumin (BSA) solution (3% w/v; Sigma-Aldrich) in Tris-buffered saline (TBS; Fisher, Pittsburgh, PA, USA) containing 0.01% Tween-20 (TBS-T; Sigma-Aldrich) for 2 h at room temperature. The membrane was treated with a primary antibody [an anti-Aβ antibody (6E10, 1:2,000; Covance, Princeton, NJ, USA), an anti-amyloid

oligomer antibody (A11, 1:1,000; Thermo Scientific), or an anti-amyloid fibril antibody (OC, 1:1,000; Millipore, Billerica, MA, USA)] in a solution of 2% BSA (w/v, in TBS-T) for 4 h at room temperature and then incubated with a horseradish peroxidase-conjugated goat anti-mouse secondary antibody (1:5,000; Cayman Chemical) for 6E10-treated membranes or a horseradish peroxidase-conjugated anti-rabbit secondary antibody (1:2,500; Promega) for the membranes treated with A11 or OC in 2% BSA in TBS-T solution (w/v) for 1 h at room temperature. A self-made ECL solution (2.5 mM luminol, 0.20 mM *p*-coumaric acid, and 0.018% H_2O_2 in 100 mM Tris, pH 8.6) was used to visualize the results on a ChemiDoc MP Imaging System (BioRad, Hercules, CA, USA).

Electrospray Ionization-Ion Mobility-Mass Spectrometry (ESI-IM-MS). (i) [$A\beta_{40}$ (100 μM) with **Ir-Me**, **Ir-H**, **Ir-F**, or **Ir-F2** (500 μM)], (ii) [α-Syn or hIAPP (100 μM) with **Ir-F** (100 μM)], and (iii) [$A\beta_{28}$ and/or $A\beta_{42}$ (100 μM) with **Ir-F** (100 μM)] upon 10 min exposure to 1 sun light were incubated in H_2O at 37 °C for 1 h without agitation. The incubated samples were diluted by 10 fold and then injected into a mass spectrometer. The capillary voltage, sampling cone voltage, and source temperature were set to 2.8 kV, 70 V, and 60 °C, respectively. The backing pressure was adjusted to 3.2 mbar. Ion mobility wave height and velocity were adjusted to 10 V and 450 m s^{-1}, respectively, and gas flow for the helium cell and ion mobility cell was set to 120 and 30 mL min^{-1}, respectively. Tandem MS (MS^2) analysis was additionally performed on the +3-charged [$A\beta_{40}$ + **Ir-F'**] (*m/z* = 1656) and singly oxidized $A\beta_{40}$ (*m/z* = 1449). The ESI parameters and experimental conditions were same as above. Collision-induced dissociation was conducted by applying the collision energy in the trap. More than 200 spectra were obtained for each sample and averaged for analysis.

Aβ Aggregation Experiments. Aβ experiments were conducted as previously published.[8] Aβ peptides were dissolved with ammonium hydroxide (NH_4OH, 1% v/v, aq), aliquoted, lyophilized, and stored at -80 °C. A stock solution (*ca.* 200 μM) was prepared by re-dissolving Aβ with NH_4OH (1% w/v, aq, 10 μL) followed by dilution with H_2O. The peptide concentration in solution was determined by measuring the absorbance of the solution at 280 nm (ε = 1450 M^{-1} cm^{-1} for $A\beta_{40}$; ε = 1490 M^{-1} cm^{-1} for $A\beta_{42}$ and $A\beta_{28}$). (i) For the experiments in cell growth media, $A\beta_{40}$ (100 μM) was first treated with **Ir-F** (100 μM; 5% v/v final DMSO concentration) in H_2O followed by exposure to 1 sun light for 10 min. The resultant samples were incubated at 37 °C for 1 h without agitation. The samples were then diluted by two fold with cell growth media or H_2O (*vide infra*) and incubated for additional 24 h at 37 °C without agitation. (ii) For the inhibition experiments, Aβ (25 μM; H_2O) was first treated with Ir(III) complexes (*i.e.*, **Ir-Me**, **Ir-H**, **Ir-F**, or **Ir-F2**; 250 μM; 5% v/v final DMSO concentration) followed by exposure to 1 sun light for 10 min. The resulting samples were incubated at 37 °C for 24 h with constant agitation. (iii) For the disaggregation experiments, Aβ (25 μM; H_2O) was initially incubated at 37 °C for 2, 4, or 24 h with steady agitation. Ir(III) complexes (250 μM; 5% v/v final DMSO concentration) were added to the preincubated Aβ samples with or without 1 sun light for 10 min followed by an additional 4 h of incubation at 37 °C with constant agitation. (iv) For $A\beta_{28}$ experiments, $A\beta_{28}$ (50 μM; H_2O) was treated with **Ir-F** (10 μM; 1% v/v final DMSO concentration) and incubated at 37 °C for 1 h with steady agitation. $A\beta_{40}$ or $A\beta_{42}$ (20 μM; H_2O) was treated to the $A\beta_{28}$–**Ir-F'** sample followed by exposure to 1 sun light for 10 min. The resulting samples were incubated at 37 °C for 1 h with constant agitation. For the experiments under anaerobic conditions, all samples were prepared

following the same procedure described above for the aerobic samples in a N_2-filled glovebox.

Gel Electrophoresis with Western Blotting (Gel/Western Blot). The resultant Aβ species from both inhibition and disaggregation experiments were analyzed by gel electrophoresis followed by Western blotting (gel/Western blot) using an anti-Aβ antibody (6E10), an anti-$A\beta_{40}$ antibody (Abcam, Cambridge, UK), anti-$A\beta_{42}$ antibody (Merck KGaA), or an anti-methionine (Met) sulfoxide antibody (Cayman Chemical, Ann Arbor, MI, USA). Each sample (10 μL) was separated using a 10–20% gradient Tris-tricine gel (Invitrogen, Grand Island, NY, USA). The gel was transferred to a nitrocellulose membrane and blocked with a BSA solution (3% w/v; Sigma-Aldrich) in TBS (Fisher) containing 0.1% Tween-20 (TBS-T; Sigma-Aldrich) for 3 h at room temperature. The membrane was treated with either an anti-Aβ antibody (6E10; 1:2,000), an anti-$A\beta_{40}$ antibody (1 μg mL^{-1}), an anti-$A\beta_{42}$ antibody (1:2,000), or an anti-methionine (Met) sulfoxide antibody (1:200) in a solution of 2% BSA (w/v, in TBS-T) for 4 h at room temperature and then incubated with a horseradish peroxidase-conjugated goat anti-mouse secondary antibody (Cayman Chemical, Ann Arbor, MI, USA; 1:5,000) for 6E10- and an anti-$A\beta_{40}$ antibody-treated membranes or a horseradish peroxidase-conjugated anti-rabbit secondary antibody (Promega, Madison, WI, USA; 1:2,500) for the membranes treated with an anti-Met sulfoxide antibody and an anti-$A\beta_{42}$ antibody in 2% BSA in TBS-T solution (w/v) for 1 h at room temperature. A self-made ECL solution (2.5 mM luminol, 0.20 mM *p*-coumaric acid, and 0.018% H_2O_2 in 100 mM Tris, pH 8.6) was used to visualize the results on a ChemiDoc MP Imaging System (BioRad).

Transmission Electron Microscopy (TEM). Samples for TEM were prepared following the previously reported methods.[8] Glow discharged grids (Formvar/Carbon 300-mesh; Electron

Microscopy Sciences, Hatfield, PA, USA) were treated with the resultant Aβ, α-Syn, or hIAPP (5 μL) for 2 min at room temperature. Excess sample was removed with filter paper and the grids were washed with H_2O three times. Each grid was stained with uranyl acetate (1% w/v H_2O; 5 μL) for 1 min. Uranyl acetate was blotted off and grids were dried for 20 min at room temperature. Images of samples were taken by a JEOL JEM-2100 (200 kV, 25,000× magnification) or a Tecnai F30 (FEI; 200 kV, 29,000× magnification) transmission electron microscope.

Inductively Coupled Plasma-Mass Spectrometry (ICP-MS). Mouse Neuro-2a (N2a) neuroblastoma cells were purchased from the American Type Cell Collection (ATCC, Manassas, VA, USA). Cells were maintained in the media containing 50% DMEM (GIBCO, Grand Island, NY, USA) and 50% opti-MEM (GIBCO), supplemented with 5% (v/v) fetal bovine serum (FBS; GIBCO), 1% (v/v) L-glutamine (GIBCO), 100 U/mL penicillin, and 100 mg/mL streptomycin (GIBCO). The cells were grown in a humidified atmosphere with 5% CO_2 at 37 °C. $A\beta_{40}$ (final concentration, 20 μM) was added with **Ir-F** (final concentration, 5 μM) exposed to 1 sun light for 10 min, and incubated for 24 h at 37 °C. The resultant Aβ species were treated with the N2a neuroblastoma cells. After 24 h incubation, the cells were washed twice with PBS. RIPA lysis buffer (50 mM Tris, 150 mM NaCl, 0.1% SDS, 1% sodium deoxycholate, and 1% Triton X-100; pH 7.5) containing a protease inhibitor cocktail (Sigma-Aldrich) and phenylmethylsulfonyl fluoride (PMSF; Sigma-Aldrich) was added to cells to obtain cell lysates after centrifugation (16,000 rpm, 10 min). The contents of Ir in the resultant samples were determined by ICP-MS.

Cytotoxicity Studies. The N2a neuroblastoma cells were seeded in a 96 well plate (15,000 cells in 100 μL per well). (i) To identify the cytotoxicity of Ir(III) complexes, the cells were treated with

their various concentrations (5, 10, 25, 50, or 100 μM; 1% v/v final DMSO concentration) and incubated for 24 h. (ii) For the experiments with Aβ, Aβ ($Aβ_{40}$ or $Aβ_{42}$; final concentration, 20 μM) was added with the Ir(III) complexes (final concentration, 5 μM) exposed to 1 sun light for 10 min, and incubated for 24 h at 37 °C. The resultant Aβ species were treated with cells. After 24 h incubation, MTT [25 μM; 5 mg mL^{-1} in PBS (pH 7.4, GIBCO)] was incubated to each well and the plate was incubated for 3 h at 37 °C. Formazan produced by the cells was solubilized by addition of an acidic solution of *N*,*N*-dimethylformamide (DMF, 50% v/v, aq, pH 4.5) and sodium dodecyl sulfate (SDS, 20% w/v) overnight at room temperature in the dark. The absorbance was measured at 600 nm using a microplate reader. Cell viability was calculated relative to cells containing an equivalent amount of DMSO. Error bars were calculated as standard errors of mean (S.E.M.) from three independent experiments. For comparisons between two groups, Student's two-tailed unpaired *t* test was employed. Statistical difference was considered significant at $*P < 0.05$.

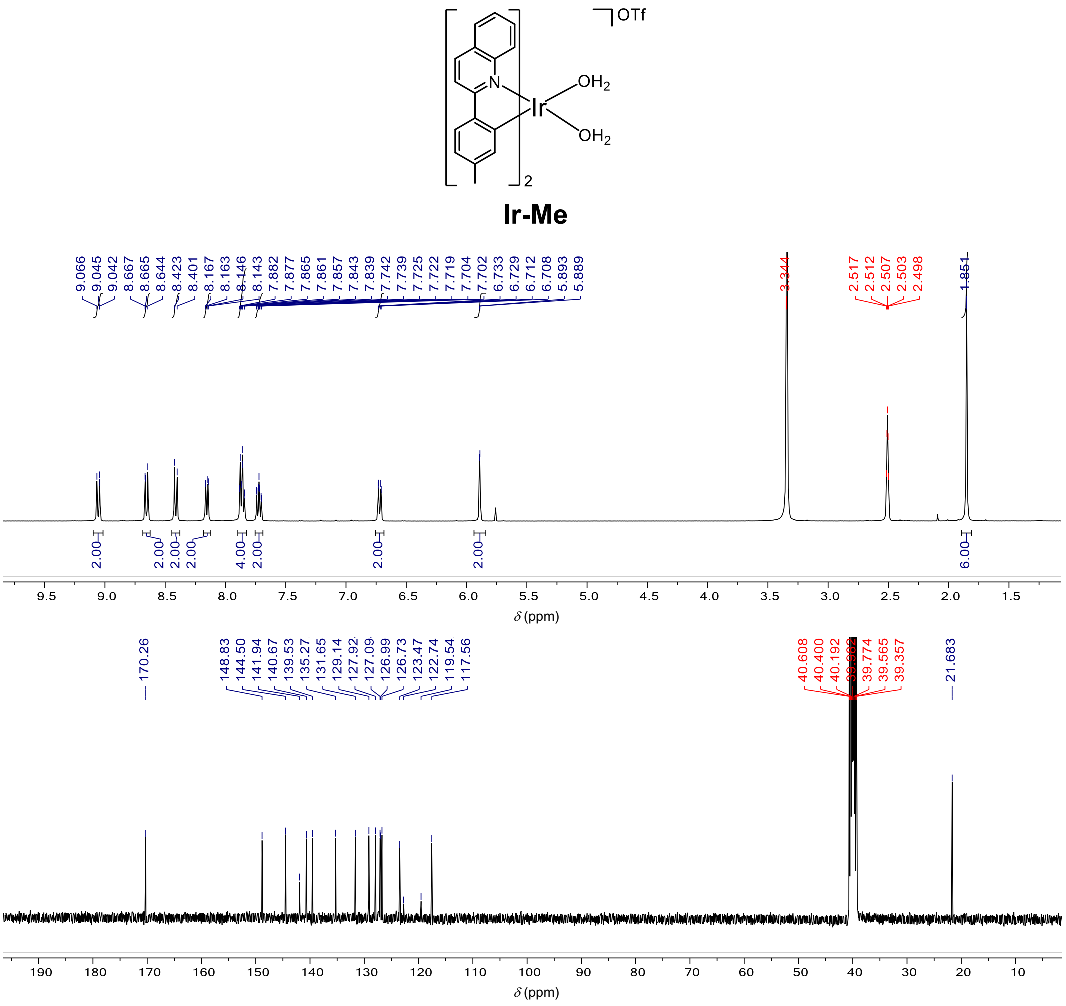

Fig. S1 ^{1}H and ^{13}C NMR spectra of **Ir-Me** in DMSO-d_6.

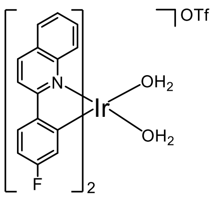

Ir-F

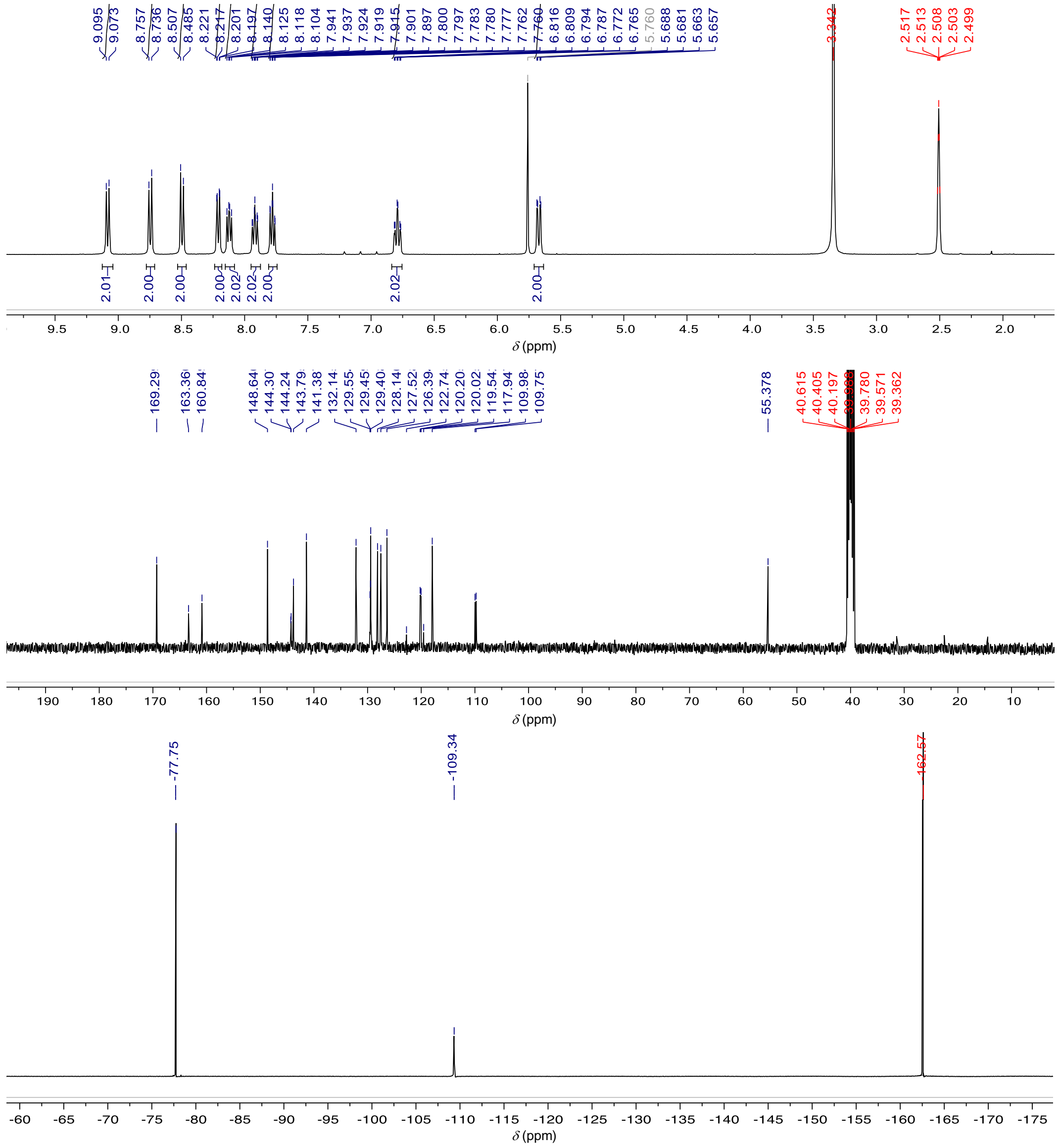

Fig. S2 ^{1}H, ^{13}C, and ^{19}F NMR spectra of **Ir-F** in DMSO-d_6.

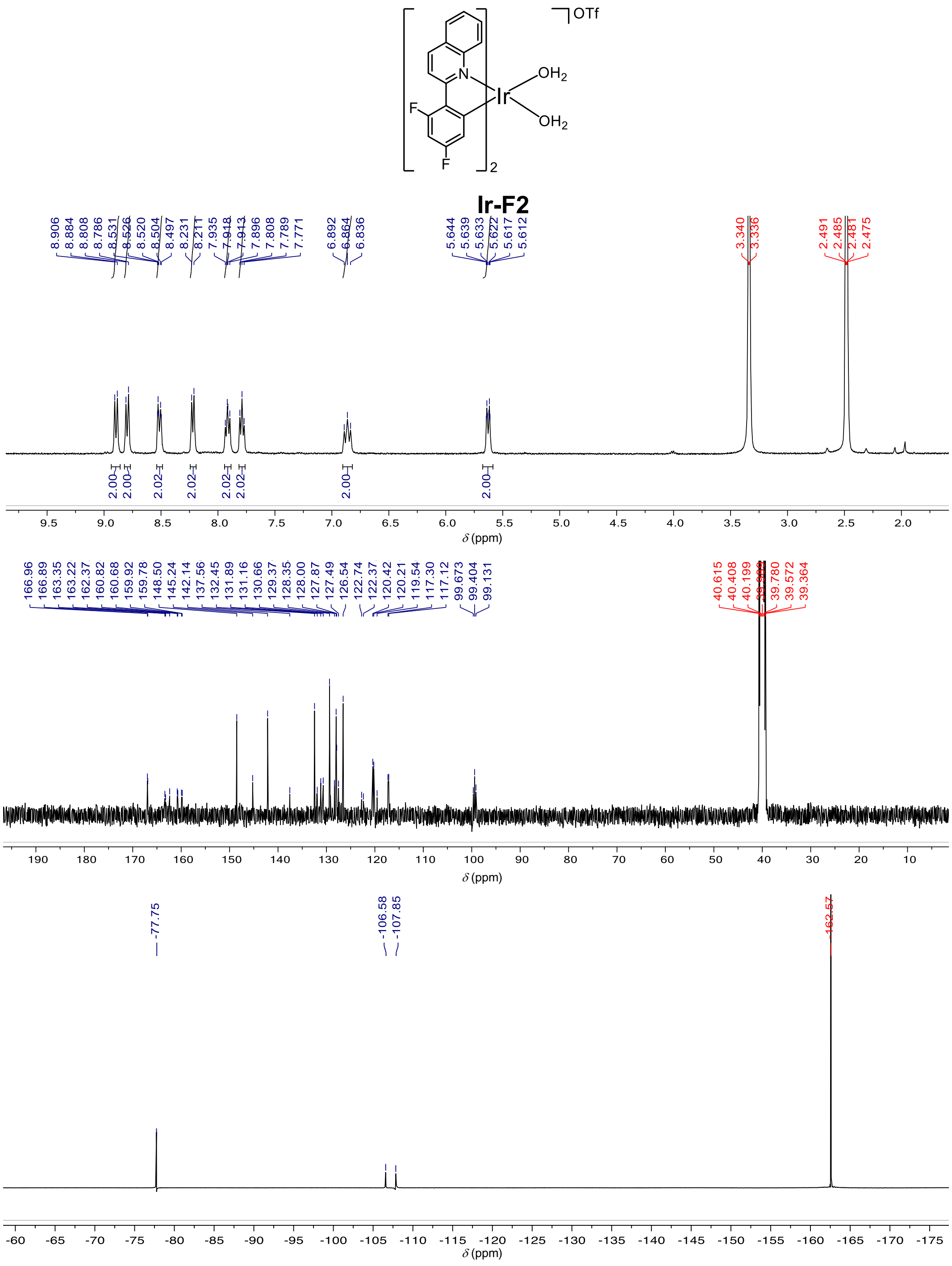

Fig. S3 ^{1}H, ^{13}C, and ^{19}F NMR spectra of **Ir-F2** in DMSO-d_6.

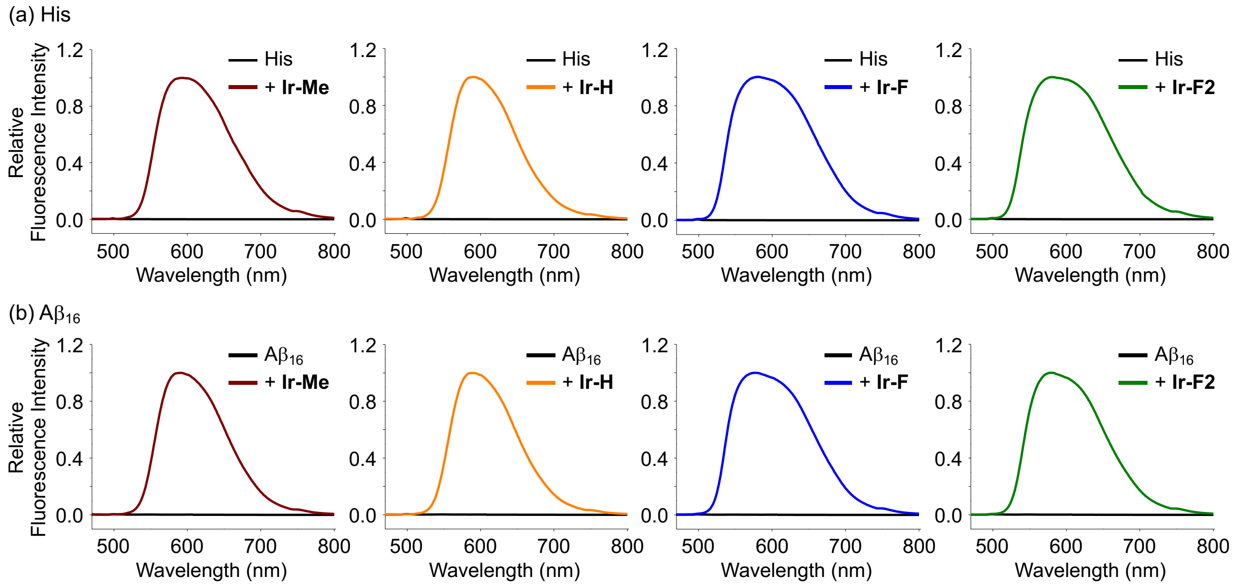

Fig. S4 Fluorescence responses of Ir(III) complexes to (a) His and (b) Aβ in DMSO. Conditions: [Ir(III) complex] = 20 μM; [His or $A\beta_{16}$] = 100 μM; room temperature; no agitation.

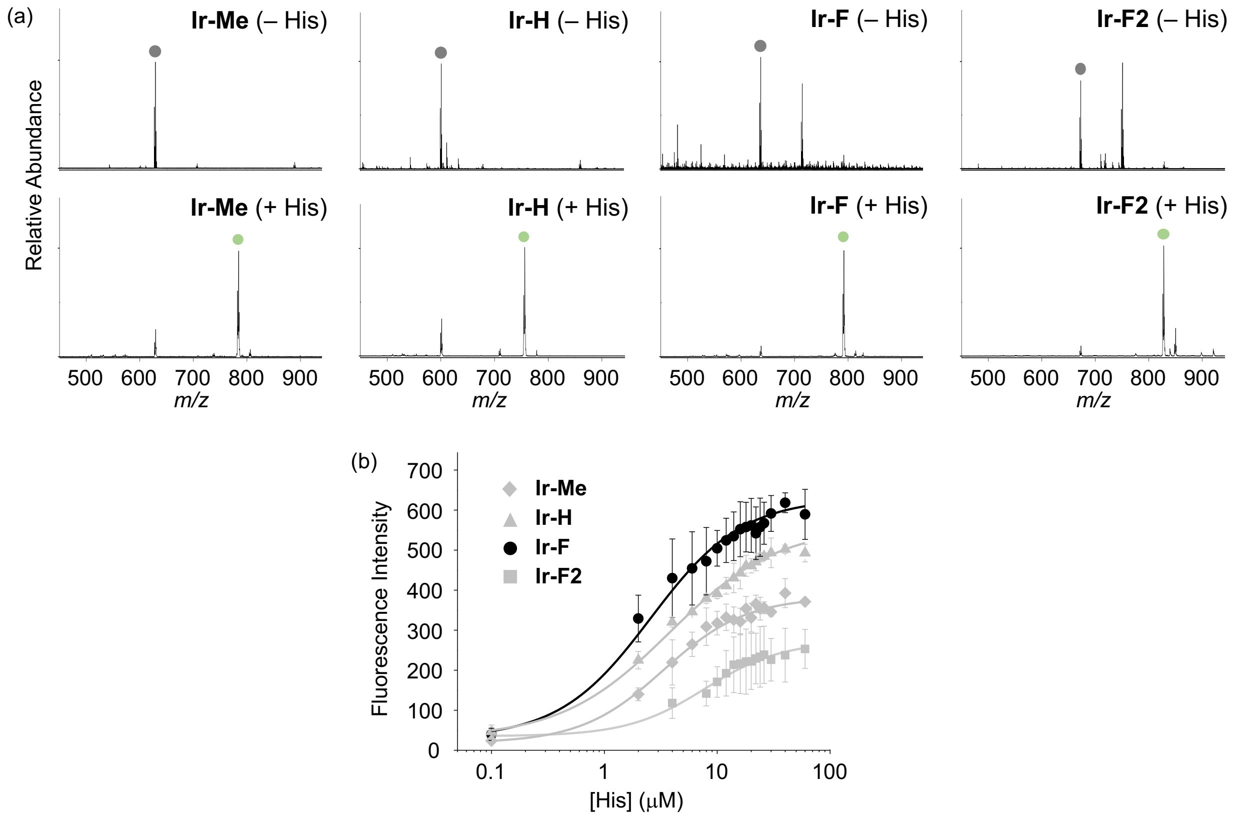

	**Ir-Me**	**Ir-H**	**Ir-F**	**Ir-F2**
K_d (x 10^{-6} M)	4.2	3.5	2.7	11

Fig. S5 His binding of Ir(III) complexes. (a) ESI-MS analysis of Ir(III) complexes (gray dots; top) with and without His. The complex formation between His and Ir(III) complexes that do not contain two H_2O molecules on the Ir(III) center [Ir(III) complexes - $2H_2O$] was observed (light green dots; bottom). Conditions: [Ir(III) complex] = 20 μM; [His] = 200 μM; room temperature; 2 h; no agitation. (b) His binding affinities of Ir(III) complexes. Dissociation constants (K_d) were determined through His titration experiments. Conditions: [Ir(III) complex] = 2 μM; [His] = 0-60 μM; room temperature; 2 h; no agitation.

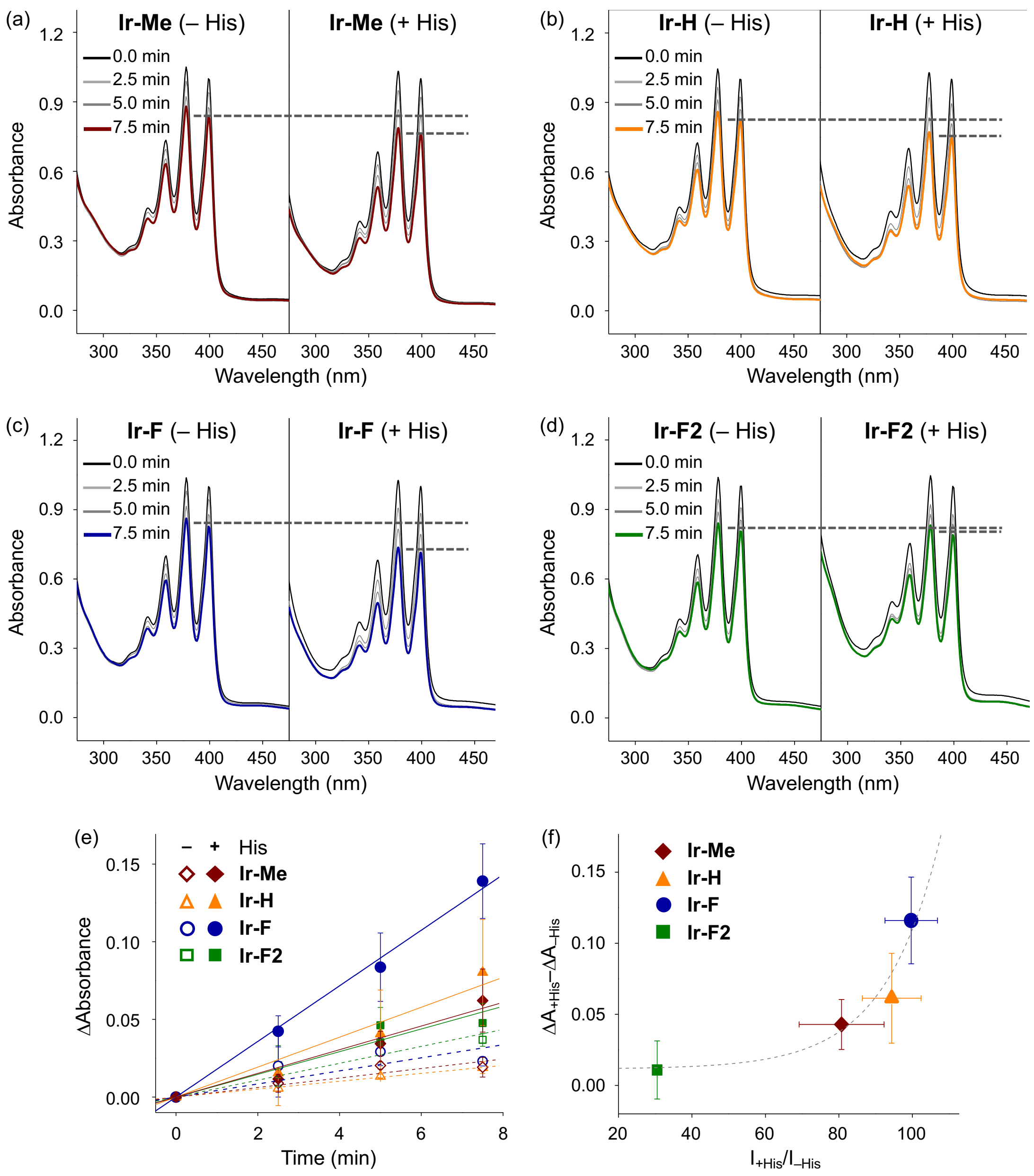

Fig. S6 Analysis of the amount of singlet oxygen ($^1O_2$) generated by Ir(III) complexes in the absence and presence of His. (a-d) Time-dependent absorbance of 9,10-anthracenediyl-bis(methylene)dimalonic acid (ABDA) upon $^1O_2$ generation triggered by (a) **Ir-Me**, (b) **Ir-H**, (c) **Ir-F**, and (d) **Ir-F2**. (e) Absorbance attenuation of ABDA with photoactivated Ir(III) complexes. (f) Correlation between the ability to produce $^1O_2$ and the emission intensity of Ir(III) complexes upon His binding. Note that $^1O_2$ generation is closely related to the triplet-to-singlet electronic

transition of the complexes. Since $^1O_2$ generation takes place through energy transfer, it is important that the energy donor (*i.e.*, Ir(III) complex) can efficiently emit the photon (phosphorescence) by transferring its energy to the energy acceptor (ground state O_2). Conditions: [Ir(III) complex] = 10 μM; [His] = 0 or 10 μM; [ABDA] = 100 μM; room temperature; 40% of 1 sun light.

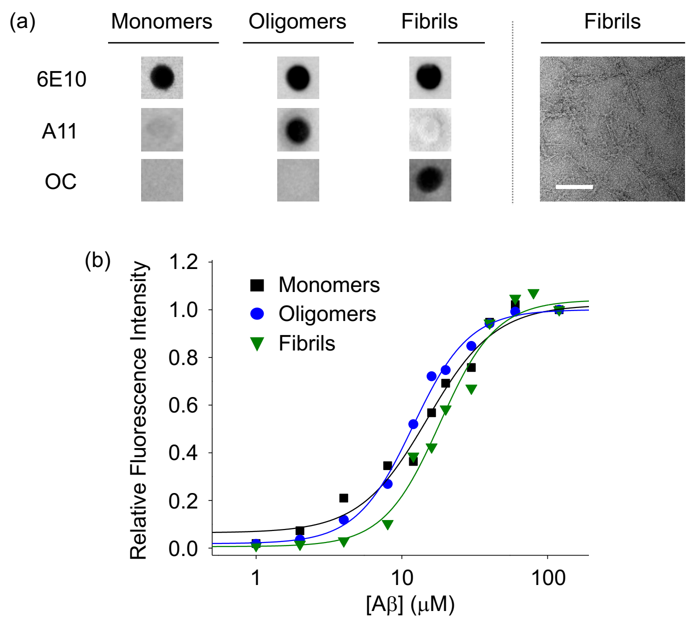

	Monomers	Oligomers	Fibrils
K_d	1.6 x 10^{-4} M	2.6 x 10^{-4} M	7.1 x 10^{-4} M

Fig. S7 Binding affinities of **Ir-F** towards different Aβ species. (a) Analysis of Aβ species generated upon incubation for 0, 2, and 48 h, monitored by the dot blot assay employing an anti-Aβ antibody (6E10), an anti-amyloid oligomer antibody (A11), and an anti-amyloid fibril antibody (OC) and TEM (right; scale bar = 200 nm) [(Monomers) $A\beta_{16}$, 0 h incubation; (Oligomers) $A\beta_{42}$, 2 h incubation; (Fibrils) $A\beta_{42}$, 48 h incubation]. $A\beta_{16}$ and $A\beta_{42}$ were used to prepare monomers and aggregates, respectively. Conditions for the dot bolt assay and TEM: [$A\beta_{16}$ or $A\beta_{42}$] = 50 μM; 37 °C; 0, 2, or 48 h. (b) Fluorescence titration results for determining binding affinities (dissociation constant, K_d) of **Ir-F** with different Aβ species. Conditions for fluorescence titration experiments: [**Ir-F**] = 4 μM; [$A\beta_{16}$ or $A\beta_{42}$] = 0-120 μM. Note that Aβ species prepared by 2 and 48 h incubation would contain a mixture of Aβ aggregates, since it is difficult to prepare homogeneous certain-sized Aβ aggregates due to the heterogenous nature of Aβ aggregation.[9]

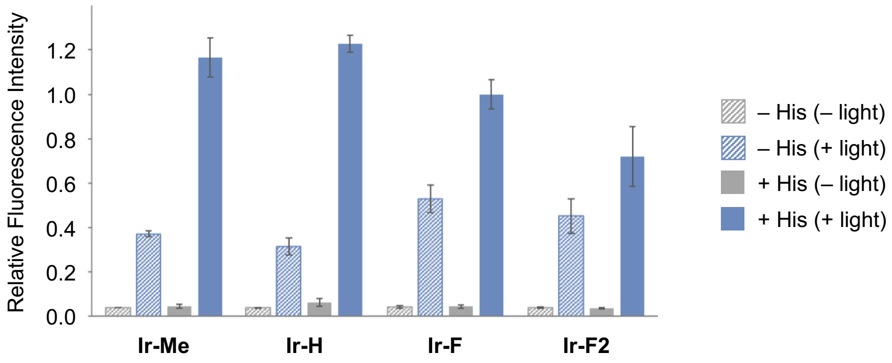

Fig. S8 Determination of the amount of superoxide anion radicals ($O_2^{\bullet-}$) produced by Ir(III) complexes with and without His, monitored by the DHR123 assay. Conditions: [Ir(III) complex] = 10 μM; [His] = 100 μM; [DHR123] = 10 μM; room temperature; 1 sun light for 5 min (for the samples treated with light).

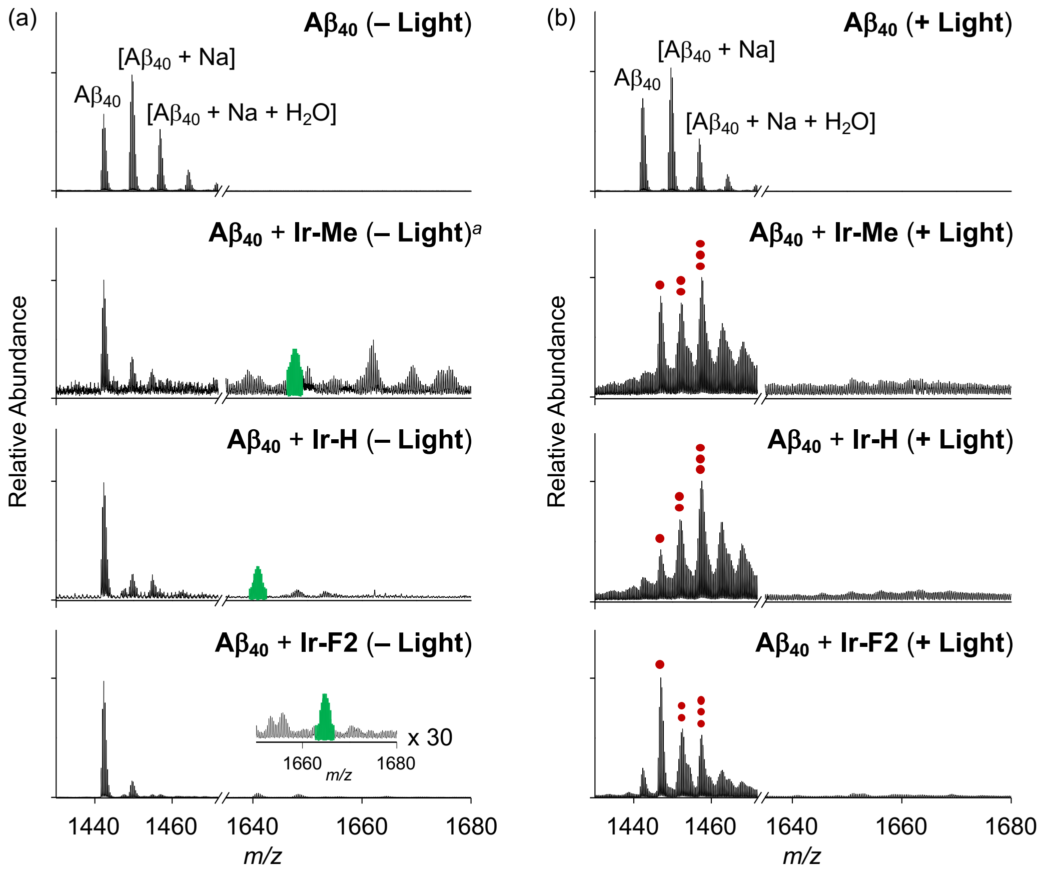

Fig. S9 ESI-MS spectra of +3-charged Aβ40 treated with **Ir-Me**, **Ir-H**, and **Ir-F2** in the (a) absence and (b) presence of light. The peaks [shown in (a)] corresponding to the complexes composed of Aβ40 and Ir(III) complexes are indicated in green. The peaks [shown in (b)] corresponding to oxidized Aβ40 species are highlighted with red dots. The number of red dots presents the number of oxygen atoms incorporated into Aβ40. Conditions: [Aβ40] = 100 μM; [Ir(III) complex] = 500 μM; 37 °C; 1 h ([a]0.5 h for the sample of Aβ40 with **Ir-Me** without light treatment); no agitation; 1 sun light for 10 min (for the samples treated with light); aerobic conditions.

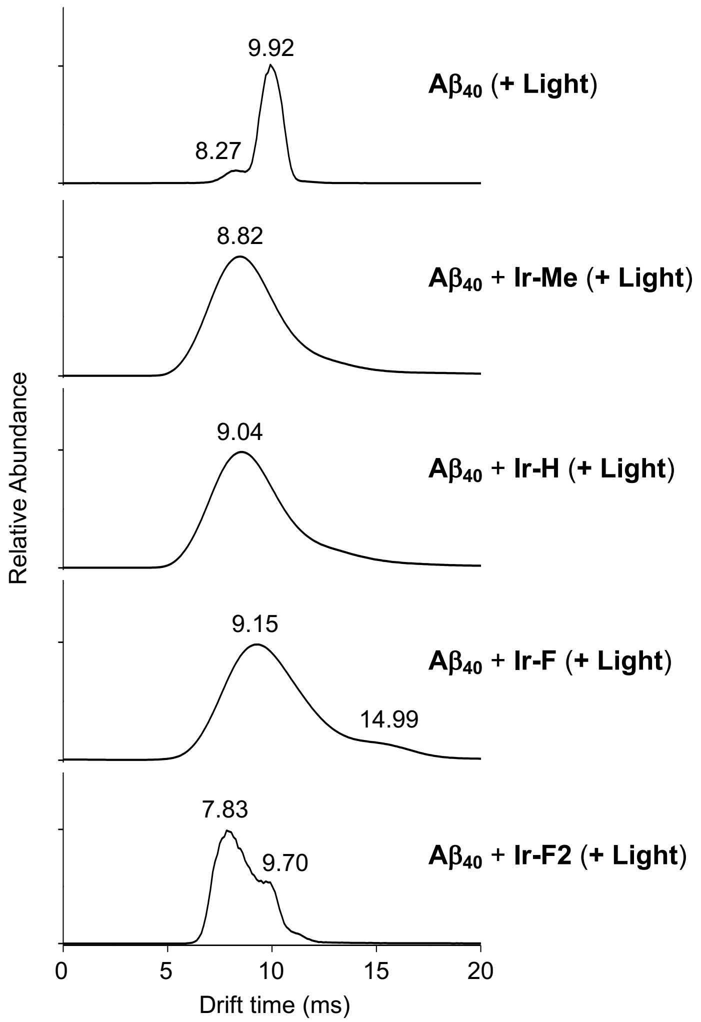

Fig. S10 IM-MS spectra of +3-charged $A\beta_{40}$ incubated with and without Ir(III) complexes. Arrival time distributions (ATDs) for nonoxidized (top; the "$A\beta_{40}$ + Light" sample) and singly oxidized $A\beta_{40}$ generated upon treatment of light-activated Ir(III) complexes were detected.

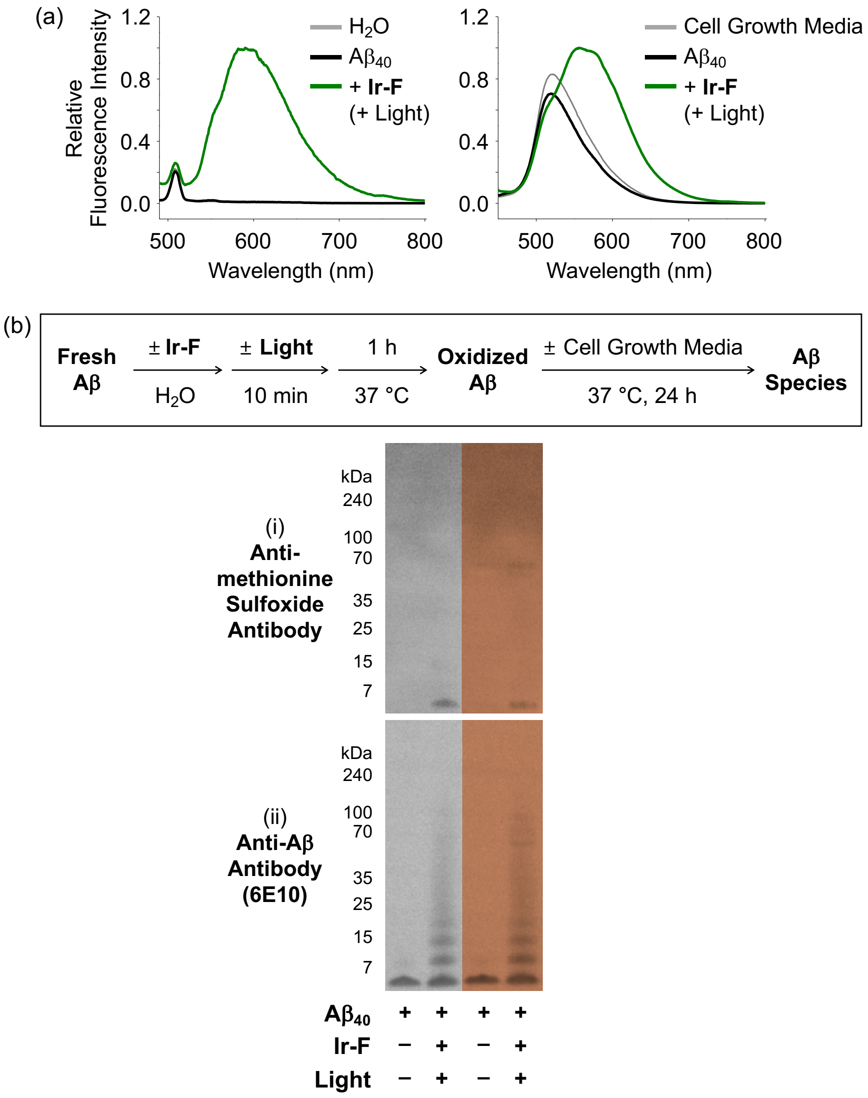

Fig. S11 Analysis of oxidized Aβ species produced by photoactivated **Ir-F**. (a) Fluorescence intensities of the resultant Aβ samples formed by 24 h treatment of **Ir-F** after light activation for 10 min in H_2O (left) and cell growth media (right). Conditions (final concentration): [$A\beta_{40}$] = 2 μM; [**Ir-F**] = 20 μM; 37 °C; 24 h; 1 sun light for 10 min (for the samples treated with light); aerobic conditions. (b) Gel/Western blots of Aβ species generated by photoactivated **Ir-F** using an (i) anti-Met sulfoxide antibody and an (ii) anti-Aβ (6E10) antibody. Aβ samples were treated with **Ir-F** and irradiated with light in H_2O followed by additional incubation in either H_2O (gray) or cell

growth media (orange). Conditions (final concentration): [Aβ_{40}] = 50 μM; [**Ir-F**] = 50 μM; 37 °C; no agitation; 1 sun light for 10 min (for the samples treated with light); aerobic conditions.

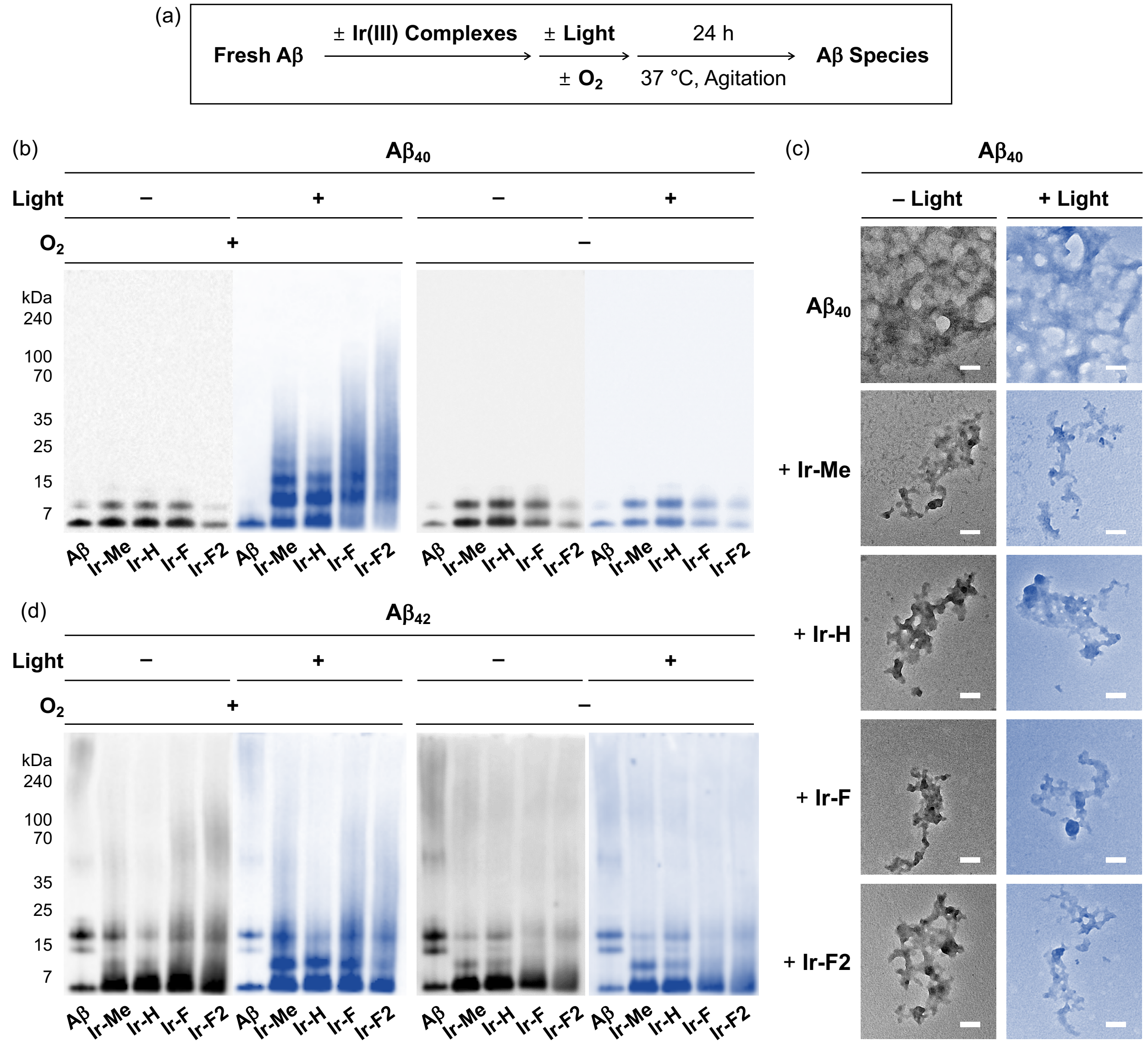

Fig. S12 Influence of Ir(III) complexes on the formation of Aβ aggregates. (a) Scheme of the experiments. Analyses of the resultant (b) $A\beta_{40}$ and (d) $A\beta_{42}$ species produced from various experimental conditions by gel/Western blot with an anti-Aβ antibody (6E10). Conditions: [Aβ] = 25 μM; [Ir(III) complex] = 250 μM; 1 sun light for 10 min (for the samples treated with light). (c) TEM images of the $A\beta_{40}$ aggregates produced from (b) (scale bar = 100 nm).

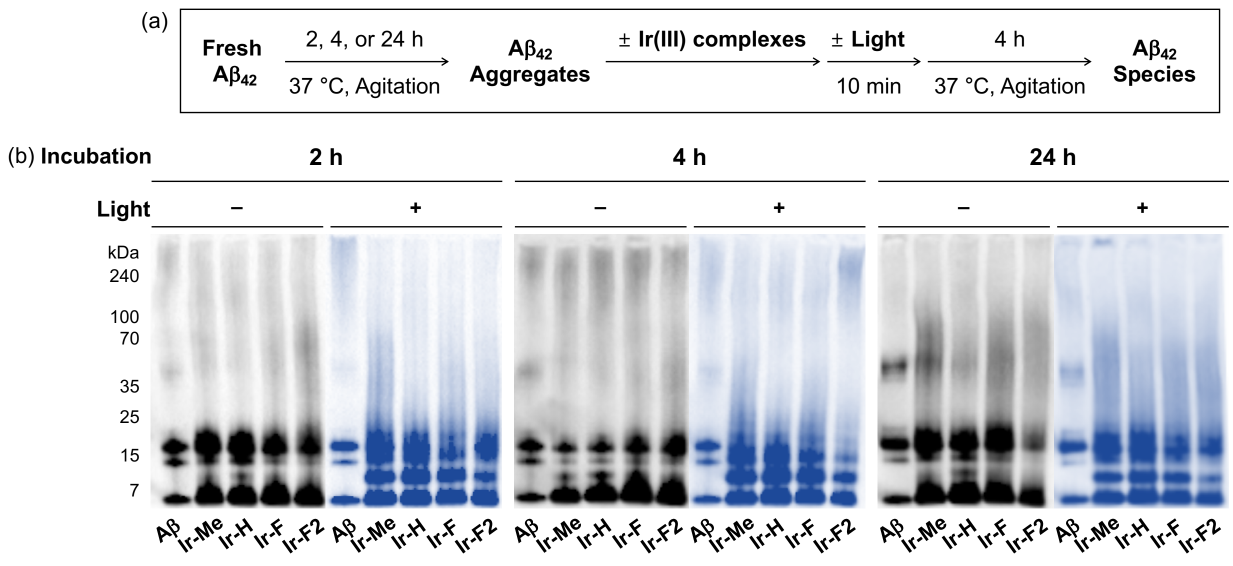

Fig. S13 Impact of Ir(III) complexes on the disassembly of preformed $A\beta_{42}$ aggregates produced at various incubation time points (*i.e.*, 2, 4, and 24 h). (a) Scheme of the experiments. (b) Analysis of the resultant $A\beta_{42}$ species by gel/Western blot with an anti-Aβ antibody (6E10). Conditions: $[A\beta_{42}]$ = 25 μM; [Ir(III) complex] = 250 μM; 1 sun light for 10 min (for the samples treated with light).

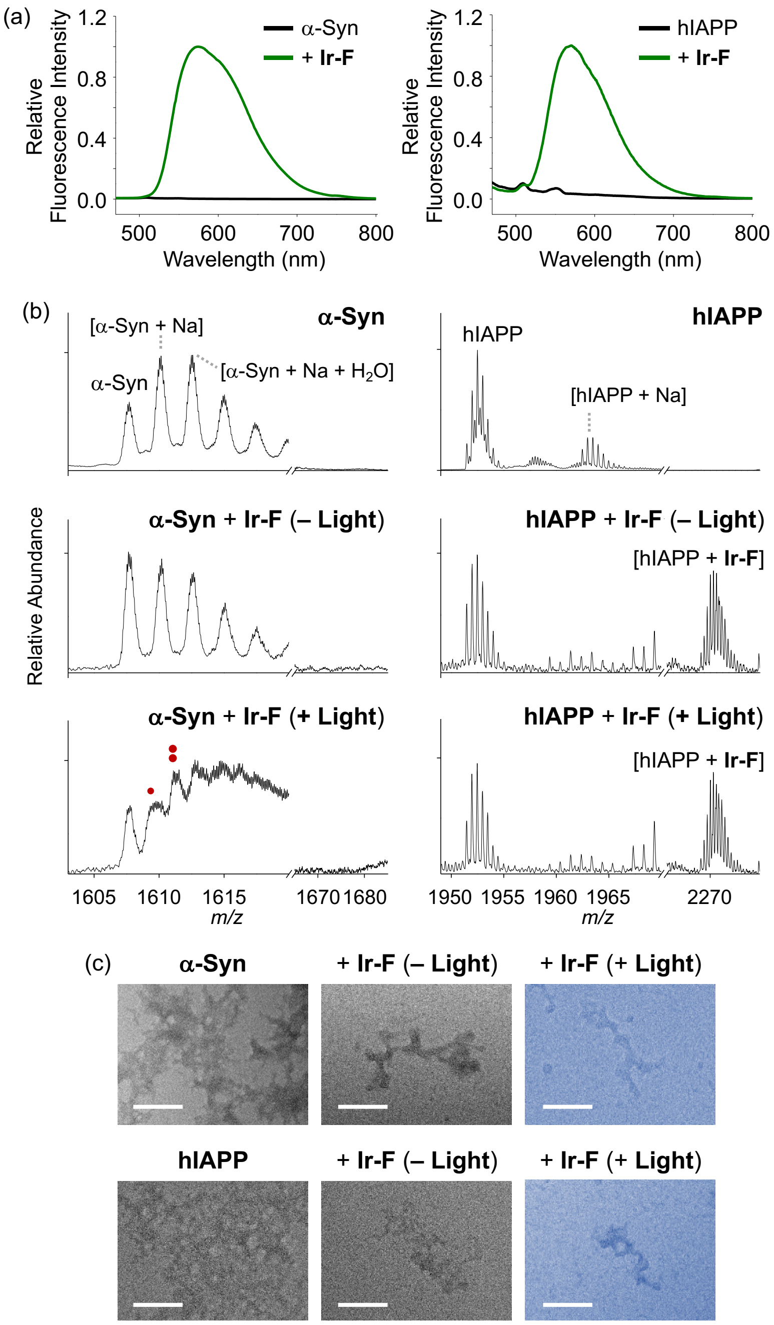

Fig. S14 Ability of **Ir-F** to modify other amyloidogenic peptides, *i.e.*, α-Syn and hIAPP, and control their aggregation pathways. (a) Fluorescence responses of **Ir-F** to α-Syn (left) and hIAPP (right). Conditions: [α-Syn or hIAPP] = 100 μM; [**Ir-F**] = 20 μM; room temperature; no agitation. (b) ESI-MS spectra of **Ir-F**-incubated +9-charged α-Syn or +2-charged hIAPP with and without

light. The peaks corresponding to oxidized peptides are indicated with red dots. The number of red dots presents the number of oxygen atoms incorporated into the peptides. Charges are omitted in the MS spectra. Conditions: [α-Syn or hIAPP] = 100 μM; [**Ir-F**] = 500 μM; 37 °C; 1 h; no agitation; 1 sun light for 10 min (for the samples treated with light); aerobic conditions. (c) TEM analyses of the resultant peptide species generated upon incubation with **Ir-F** (scale bar = 200 nm). Conditions: [α-Syn or hIAPP] = 25 μM; [**Ir-F**] = 125 μM; 37 °C; 24 h; constant agitation; 1 sun light for 10 min (for the samples treated with light); aerobic conditions.

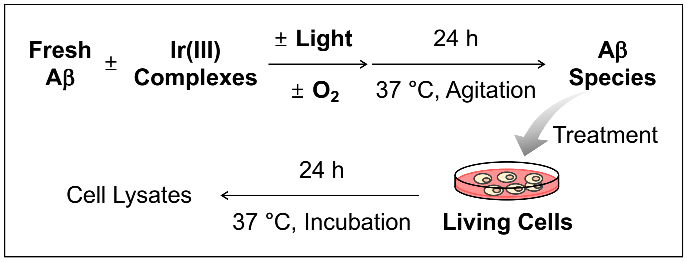

	$A\beta_{40}$	$A\beta_{40}$ + **Ir-F** (+ Light)	**Ir-F** (+ Light)
Ir (μg L^{-1})	0.17	39	34

Fig. S15 Cellular uptake of Aβ species generated by light-activated **Ir-F**. The N2a cell lysates treated with the resultant Aβ samples, containing a mixture of photooxidized Aβ, the complex between **Ir-F** and Aβ, and **Ir-F**, were analyzed by ICP-MS. Conditions (final concentration): [Aβ] = 20 μM; [**Ir-F**] = 5 μM.

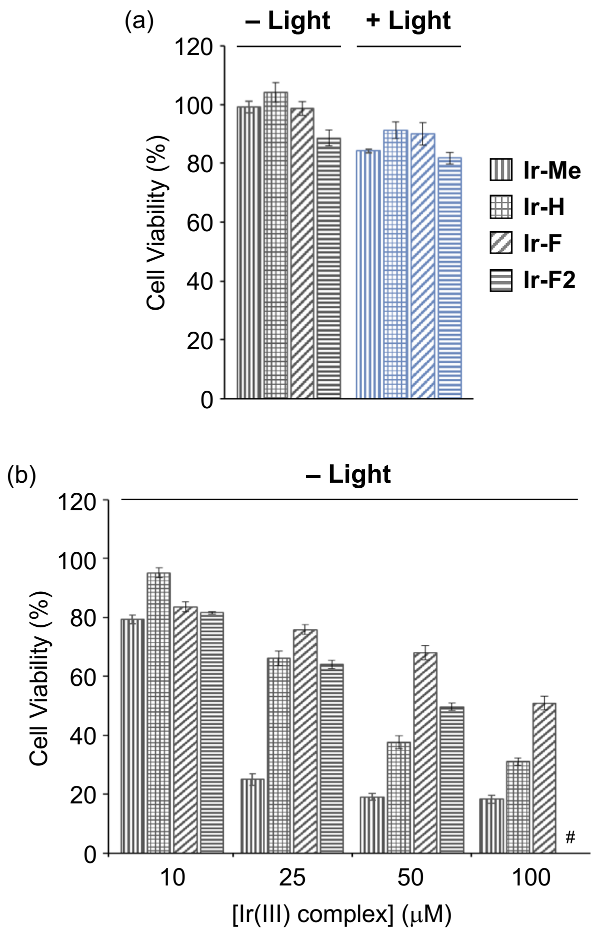

Fig. S16 Viability of N2a cells with Ir(III) complexes. (a) Cell viability of Ir(III) complexes (5 μM) in the absence and presence of light. (b) Survival of the cells treated with different concentrations of Ir(III) complexes (10-100 μM; #the value was not obtained due to limited solubility) in the absence of light exposure. Cell viability (%), measured by the MTT assay, was calculated compared with cells obtained with an equivalent amount of DMSO only. Error bars represent S.E.M. from three independent experiments.

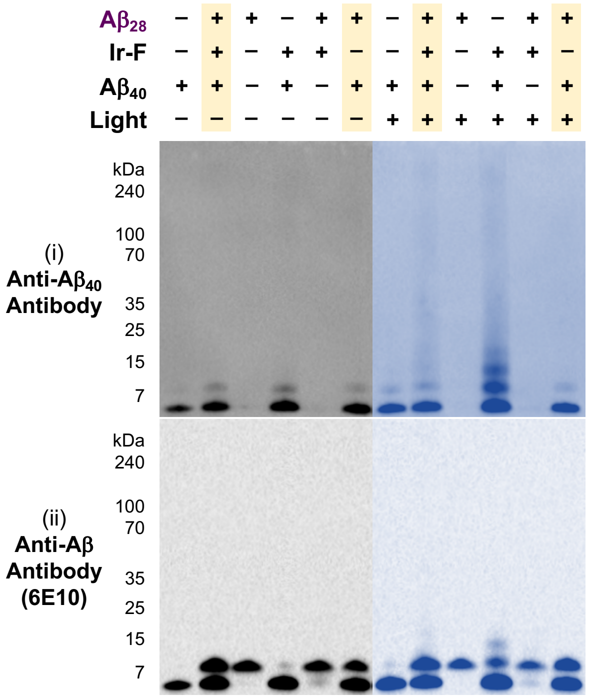

Fig. S17 Impact of **Ir-F**-preincubated $A\beta_{28}$ on $A\beta_{40}$ aggregation. Conditions: [$A\beta_{28}$] = 50 μM; [**Ir-F**] = 10 μM; [$A\beta_{40}$] = 20 μM; 37 °C; 2 h; constant agitation; 1 sun light for 10 min (for the samples treated with light); aerobic conditions.

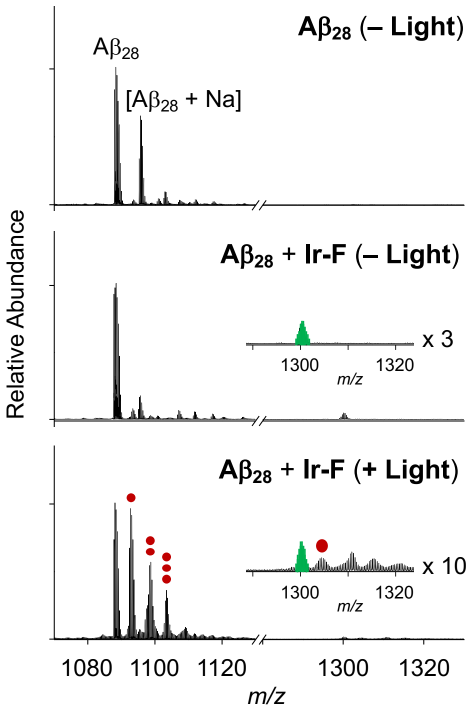

Fig. S18 ESI-MS spectra of +3-charged Aβ$_{28}$ incubated with **Ir-F** in the absence and presence of light. The peak indicated in green refers to the complex generation between Aβ$_{28}$ and **Ir-F'** (the **Ir-F** form that does not contain two H_2O molecules). The peaks corresponding to oxidized Aβ$_{28}$ species are indicated with red dots. The number of red dots presents the number of oxygen atoms incorporated into Aβ$_{28}$. Conditions: [Aβ$_{28}$] = 100 μM; [**Ir-F**] = 100 μM; 37 °C; 1 h; no agitation; 1 sun light for 10 min (for the samples treated with light); aerobic conditions.

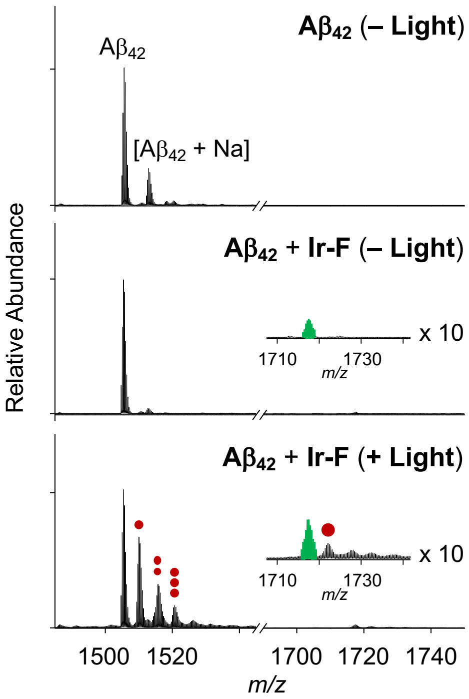

Fig. S19 ESI-MS spectra of +3-charged Aβ_{42} treated with **Ir-F** in the absence and presence of light. The peak indicated in green refers to the complex generation between Aβ_{42} and **Ir-F'** (the **Ir-F** form that does not contain two H_2O molecules). The peaks corresponding to oxidized Aβ_{42} species are indicated with red dots. The number of red dots presents the number of oxygen atoms incorporated into Aβ_{42}. Conditions: [Aβ_{42}] = 100 μM; [**Ir-F**] = 100 μM; 37 °C; 1 h; no agitation; 1 sun light for 10 min (for the samples treated with light); aerobic conditions.

References

1 D. Haddenham, C. L. Bailey, C. Vu, G. Nepomuceno, S. Eagon, L. Pasumansky and B. Singaram, *Tetrahedron*, 2011, **67**, 576–583.

2 J. Yu, K. He, Y. Li, H. Tan, M. Zhu, Y. Wang, Y. Liu, W. Zhu and H. Wu, *Dyes and Pigm.*, 2014, **107**, 146–152.

3 B. Schmid, F. O. Garces and R. J. Watts, *Inorg. Chem.*, 1994, **33**, 9–14.

4 N. D. McDaniel, F. J. Coughlin, L. L. Tinker and S. Bernhard, *J. Am. Chem. Soc.*, 2008, **130**, 210–217.

5 M. Nonoyama, *Bull. Chem. Soc. Jpn.*, 1974, **47**, 767–768.

6 J. S. Nam, M.-G. Kang, J. Kang, S.-Y. Park, S. J. C. Lee, H.-T. Kim, J. K. Seo, O.-H. Kwon, M. H. Lim, H.-W. Rhee and T.-H. Kwon, *J. Am. Chem. Soc.*, 2016, **138**, 10968–10977.

7 T.-H. Kwon, H. S. Cho, M. K. Kim, J.-W. Kim, J.-J. Kim, K. H. Lee, S. J. Park, I.-S. Shin, H. Kim, D. M. Shin, Y. K. Chung and J.-I. Hong, *Organometallics*, 2005, **24**, 1578–1585.

8 J. Kang, S. J. C. Lee, J. S. Nam, H. J. Lee, M.-G. Kang, K. J. Korshavn, H.-T. Kim, J. Cho, A. Ramamoorthy, H.-W. Rhee, T.-H. Kwon and M. H. Lim, *Chem. Eur. J.*, 2017, **23**, 1645–1653.

9 M. G. Savelieff, G. Nam, J. Kang, H. J. Lee, M. Lee and M. H. Lim, *Chem. Rev.*, 2019, **119**, 1221–1322.
